# Improvement of oxygen reduction activity and stability on a perovskite oxide surface by electrochemical potential

**DOI:** 10.1038/s41467-023-42462-5

**Published:** 2023-11-08

**Authors:** Sanaz Koohfar, Masoud Ghasemi, Tyler Hafen, Georgios Dimitrakopoulos, Dongha Kim, Jenna Pike, Singaravelu Elangovan, Enrique D. Gomez, Bilge Yildiz

**Affiliations:** 1 Laboratory for Electrochemical Interfaces, Massachusetts Institute of Technology, Cambridge, MA USA; 2 Department of Nuclear Science and Engineering, Massachusetts Institute of Technology, Cambridge, MA USA; 3 Department of Chemical Engineering, The Pennsylvania State University, University Park, PA USA; 4 Department of Materials Science and Engineering, The Pennsylvania State University, University Park, PA USA; 5 OxEon Energy, LLC, North Salt Lake, UT USA; 6 Department of Materials Science and Engineering, Massachusetts Institute of Technology, Cambridge, MA USA

**Keywords:** Fuel cells, Porous materials, Energy

## Abstract

The instability of the surface chemistry in transition metal oxide perovskites is the main factor hindering the long-term durability of oxygen electrodes in solid oxide electrochemical cells. The instability of surface chemistry is mainly due to the segregation of A-site dopants from the lattice to the surface. Here we report that cathodic potential can remarkably improve the stability in oxygen reduction reaction and electrochemical activity, by decomposing the near-surface region of the perovskite phase in a porous electrode made of La_1-x_Sr_x_Co_1-x_Fe_x_O_3_ mixed with Sm_0.2_Ce_0.8_O_1.9_. Our approach combines X-ray photoelectron spectroscopy and secondary ion mass spectrometry for surface and sub-surface analysis. Formation of Ruddlesden-Popper phase is accompanied by suppression of the A-site dopant segregation, and exsolution of catalytically active Co particles onto the surface. These findings reveal the chemical and structural elements that maintain an active surface for oxygen reduction, and the cathodic potential is one way to generate these desirable chemistries.

## Abstract (web summary)

Solid oxide fuel and electrolysis cells suffer from surface instability which challenges their performance and durability. Here, the authors report that cathodic polarization improves the electrochemical activity by formation of Ruddlesden-Popper phase, exsolution of Co, and suppression of Sr segregation.

## Introduction

Solid oxide fuel and electrolysis cells (SOFC/SOEC) have gained tremendous attention in recent years due to their promising energy storage and conversion capabilities^1–4^. Solid oxide electrochemical cells have the advantages of fuel flexibility^5^, lower carbon emissions^6^, and high efficiency at elevated operational temperatures. However, the surface instability of perovskite oxides serving as oxygen electrodes^7–13^ in SOFC/SOEC is a major challenge to the performance and durability of these devices. La_1-x_Sr_x_Co_1-x_Fe_x_O_3_ (LSCF) is one of the most widely used oxygen electrode materials in solid oxide electrochemical cells due to its high ionic and electronic conductivity, which are essential for oxygen exchange reactions^1,6,14^. The initial high efficiency of porous LSCF oxygen electrodes at operating voltages suffers from degradation over a long period of operation in both fuel and electrolysis modes^15–18^. Several studies have been conducted to understand the origin of this degradation mechanism and to improve stability and activity in LSCF electrodes^19–21^. Segregation of strontium (Sr) dopant is one of the main factors hindering long-term stability in a variety of perovskite oxide electrodes such as LSCF, La_1-x_Sr_x_MnO_3_ (LSM), and La_1-x_Sr_x_CoO_3_ (LSC)^16,17,22–25^. Sr segregation to the surface of perovskite oxide electrodes forms insulating SrO precipitates and layers that block the active sites that are vital for oxygen exchange reactions^8,26,27^. Consequently, numerous approaches have been explored to prevent the segregation of Sr dopant. Among these approaches, surface impregnation^22,28–33^ and applied cathodic polarization^34^ have shown promising potentials in the suppression of Sr segregation^17,28,35^. Applied cathodic polarization was shown to improve the electrochemical activity in LSM^36,37^, LSF^38^, and LSCF^34,39^ without requiring extra processing steps. Even though this approach improves the activity and stability of perovskite oxide electrodes, the underlying mechanism behind this phenomenon remains largely unclear, limiting the applicability of this method.

Here we reveal the mechanism by which cathodic electrochemical potential can improve the surface and electrochemical stability on a state-of-the-art model oxygen electrode, LSCF perovskite oxide mixed with Sm_0.2_Ce_0.8_O_1.9_ (SDC). We use LSCF-SDC as cathode and anode in a symmetric cell configuration on ScSZ (Sc_0.06_Zr_0.94_O_1.97_) electrolyte. A reference electrode is placed near the active electrode to separately monitor the evolution of electrochemical reactions on the cathode (oxygen reduction) and anode (oxygen evolution). We find that polarization overpotentials larger than 0.4 V lead to an improvement in the oxygen reduction reaction (ORR), which results in stability over a long period. In addition, the applied cathodic polarization improves the electrochemical stability and activity of the fuel cell consisting of LSCF-SDC composite as the cathode and NCS (NiO | Sm_0.2_Ce_0.8_O_1.9_) composite as the anode. We further show that the improved ORR activity and stability is accompanied by the formation of Ruddlesden-Popper (RP3, *n* = 3 in A_n+1_B_n_O_3n+1_, A_4_B_3_O_10_) phase, an increase in the B-site cations (Co and Fe) at the surface, and suppression of Sr segregation at the surface of LSCF-SDC. We observe a B-site deficient region in the sub-surface region of LSCF-SDC, which is attributed to the formation of the RP3 phase in that region.

Our approach combines X-ray photoelectron spectroscopy (XPS) and electrochemical impedance spectroscopy (EIS) to quantitively measure the concentration of surface elements and the oxygen exchange activity of LSCF-SDC, respectively. In addition, time of flight secondary ion mass spectrometry (ToF-SIMS, referred to as SIMS hereafter) is used to measure the depth profile of different cations.

## Results

### Electrochemical Impedance measurement

**Fig. 1:**
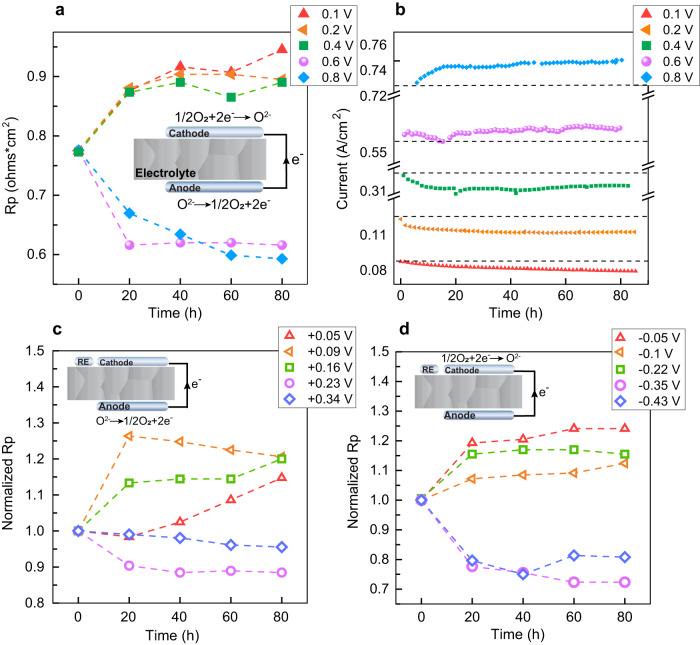
Stability of the LSCF-SDC electrodes as a function of electrochemical polarization. **a** Polarization resistance obtained from electrochemical impedance spectroscopy at open-circuit voltage as a function of time measured after polarizing the symmetric cells at different applied voltages. We have used a separate symmetric cell for each polarization voltage. **b** Current density from the symmetric cell measured at the corresponding voltages, dashed lines are plotted as a guide for the eye to show how the current density changes with respect to the initial state at that voltage. **c** Relative change of polarization resistance for oxygen evolution reaction at the anodically polarized electrode (labels indicate the overpotentials at the anode). **d** Relative change of the polarization resistance for oxygen reduction reaction at the cathodically polarized electrode (labels indicate the overpotentials at the cathode).

We measured electrochemical impedance spectroscopy (EIS) at 800 °C in ambient air on symmetric cells made of LSCF-SDC porous electrodes on ScSZ electrolyte. We fabricated the porous electrodes by screen printing method on a ScSZ electrolyte and an interlayer of SDC is placed between the electrolyte and electrodes to prevent chemical reactions between ScSZ and LSCF-SDC. A reference electrode is placed in the vicinity of working electrodes which allows us to measure the *Rp* at OCV, and the overpotential of the cathode and anode separately (see Methods section for more details on the geometry of the cells with reference electrode). The inset in Fig. 1a shows a schematic of the symmetric cell and the relevant reactions on each electrode. We used gold as the current collector on the electrode and increased the temperature to 900 °C before the EIS measurement to stabilize the gold contact. EIS measurements are performed while polarizing the cells at each corresponding voltage with 20 hour time intervals, and the current density is measured during the polarization. After each 20 hour time interval, the impedance is measured at open circuit voltage (OCV) to obtain polarization resistances (*Rp*). The sum of *Rp* measured by the reference electrode (three-electrode configuration) for the cathode and anode is equal to the total *Rp* measured without the reference electrode (two-electrode configuration) across the cell which proves the correct placement of the reference electrode (see Fig. S1 for more details). We have chosen five different polarization voltages and used a separate symmetric cell for each polarization potential.

Figure 1a shows the total polarization resistance (*Rp*) which includes ORR on the cathode and OER on the anode. *Rp* is calculated based on an equivalent circuit model reported in references^40,41^ (See Fig. S2 for details) for analyzing the impedance spectrum measured at OCV. As can be seen in Fig. 1a, polarization resistance as a function of time can be categorized into two different types. Type I is the responses of the cells operating under relatively small applied cell voltages ( < 0.6 V in total on the cell). In Type I cells, the polarization resistance increases as a function of time due to the degradation of the LSCF-SDC electrodes, and results in slower ORR and/or OER rates. On the other hand, Type II behavior is associated with the cells operating under stronger applied cell voltages (≥0.6 V). In the Type II case, polarization resistance decreases relative to the initial value; a decrease in *Rp* is associated with faster ORR and OER rates, thus indicating the activation of LSCF-SDC. A similar trend to *Rp* is observed in the current density of the cells operating at different voltages (Fig. 1b). A decrease/increase in the current density is associated with the degradation/activation of LSCF-SDC during the electrochemical polarization. The most severe current density drop is observed in 0.1 and 0.2 V cell voltages, followed by 0.4 V applied voltage. Conversely, at the applied cell voltages of 0.6 V and 0.8 V, the current density increases over time. The current density in Type II cells at 0.6 V continues to activate for the first 75 hours and then reaches a plateau for the remainder of the measurement. The current density at 0.8 V continues to activate for the entire 80 hours of measurement and *Rp* continues to decrease indicating that the cell is continuously activating under that voltage. Since the current density at 0.8 V has more increase compared to 0.6 V, the *Rp* of 0.8 V cell also activates more when compared to 0.6 V *Rp*. (See Fig. S3 for a detailed comparison between 0.6 V and 0.8 V cells)

Figure 1c and Fig. 1d show the evolution of the polarization resistance for OER on the anode and ORR on the cathode at open circuit voltage (OCV) after polarization for 20 hour time intervals. The corresponding overpotentials are measured by the reference electrode for the anode and the cathode (see the legends in Fig. 1c and Fig. 1d). The sum of two overpotentials is smaller than the total applied cell voltage because of the potential loss across the electrolyte and current collector^42^ (see supporting information for more details on validation of reference electrode placement). Here an increase/decrease in *Rp* measured on the anode (OER) and the cathode (ORR) marks the degradation/activation of LSCF-SDC electrodes similar to the total *Rp* (Fig. 1a). OER and ORR show a monotonic increase (degradation) for cells polarized at 0.1 V, 0.2 V, and 0.4 V potentials. On the other hand, for the applied cell voltages larger than 0.4 V, we observe a monotonic decrease (activation) of *Rp* for OER and ORR. Since the variation in the exact position of the reference electrode on a thin ScSZ electrolyte can create a difference in the ohmic and polarization resistances of the cathode and anode^42,43^, all results in Fig. 1c and Fig. 1d are normalized to the total *Rp* at t = 0. *Rp* decreases by 30% and 12% on the cathode and the anode, respectively, when comparing the 0 hour and 80 hour data points for the 0.6 V cell. A larger drop in *Rp* of the cathode electrode compared to the anode electrode is also observed in the 0.8 V polarized cell. Given the larger extent of improvement on the cathode, we focus on the following analysis of the material chemistry and structure on the LSCF-SDC electrodes that were polarized cathodically.

### Surface chemistry evolution on LSCF-SDC perovskite oxide

**Fig. 2:**
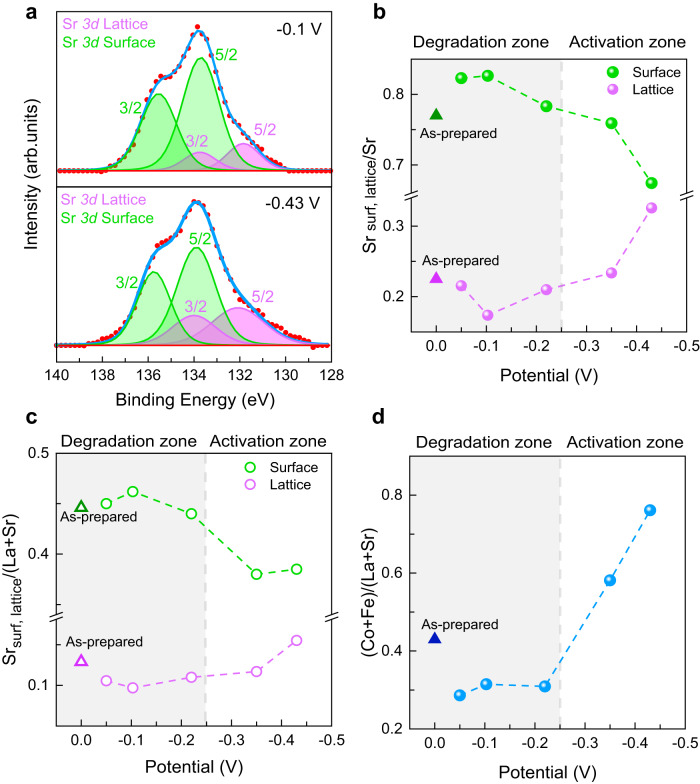
Surface chemistry of LSCF-SDC as a function of applied potential. **a** The Sr *3d* X-ray photoelectron spectra deconvolution into lattice and surface doublets for −0.1 V and −0.43 V electrodes at the top and bottom, respectively. **b** and **c** Surface and lattice concentrations of Sr normalized to total Sr and total A-site, respectively, on the surface of cathode electrodes. **d** The ratio of B-site cations to A-site cations on the surface of the cathode electrodes. The triangles show the respective quantity measured on the as-prepared electrode. The electrode potential on the x-axis for each data point represents the overpotential measured on the LSCF-SDC cathode by using a reference electrode.

We performed X-ray photoelectron spectroscopy to understand the relation between the observed electrochemical performance and the surface chemistry of the LSCF-SDC electrodes. Figure 2a shows the Sr *3d* X-ray photoelectron spectra for the LSCF-SDC electrodes polarized at −0.1 V and −0.35 V cathodic overpotentials. We deconvolute the spectrum into the lower and higher binding energy peak sets that are assigned to the lattice and non-lattice (surface) components, respectively^9,22,44^ (See Fig. S4 and Fig. S5 for deconvolution of La *3d*, Co *2p*, Co *3p*, and Fe *3p* spectra). The lattice and surface components of Sr *3d* normalized to the total Sr and total A-site cations (La and Sr) are plotted in Fig. 2b and Fig. 2c, respectively, for the cathode electrode of all cells. As can be seen, at the smaller cathodic overpotentials of −0.05 V, −0.1 V, and −0.22 V, Sr *3d* surface and lattice ratios are close to those measured on the as-prepared electrode. On the other hand, by increasing the overpotential, the contribution of the Sr *3d* lattice component becomes more prominent while the surface component of Sr *3d* decreases. The ratio of the total B-site cations (Co and Fe) to the total A-site cations (La and Sr) is shown in Fig. 2d. B/A ratio monotonically increases by increasing the cathodic overpotential with an over 60% increase in the −0.43 V electrode compared to the −0.05 V electrode. The B/A ratio also increases with an increase in the anodic overpotential by 20% in the +0.34 V electrode compared to the +0.05 V electrode (see Fig. S6). These quantitative analyses show that LSCF-SDC electrodes polarized at −0.35 V and −0.43 V have more B-site cations and less Sr precipitation at their surface, compared to the as-prepared LSCF-SDC, and the electrodes with smaller overpotentials. The shaded area in Fig. 2b, Fig. 2c, and Fig. 2d represents the degradation regime for the electrodes in this overpotential range. The activation regime includes electrodes with cathodic overpotentials of −0.35 V and −0.43 V, as shown in Fig. 1d, and the activation of these electrodes is accompanied by an increase in the B/A ratio and a decrease in the Sr surface (non-lattice) content.

**Fig. 3:**
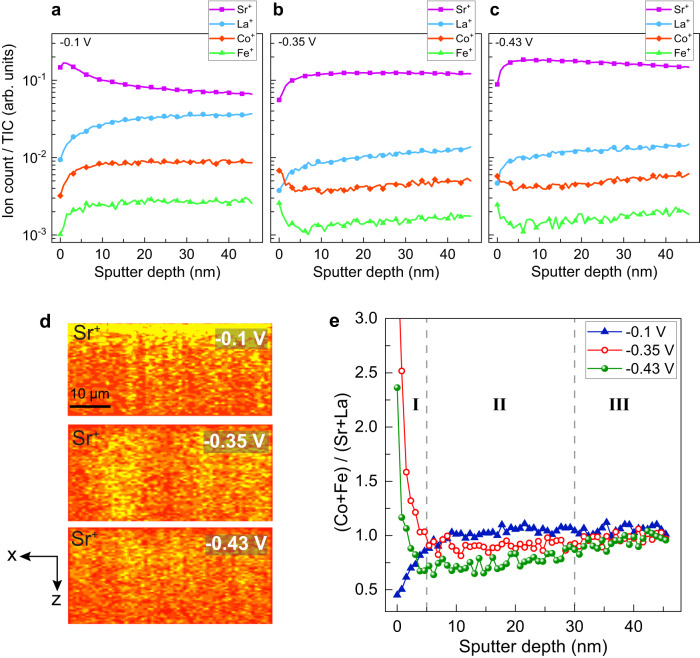
SIMS analysis of LSCF-SDC as a function of applied potential. **a**–**c** SIMS profile of different elements normalized to the total ion count as a function of sputter time for the LSCF-SDC electrodes cathodically polarized at −0.1 V, −0.35 V, and −0.43 V, respectively. **d** Reconstructed cross-section images of −0.1 V, −0.35 V, and −0.43 V electrodes using Bi_3_^++^ as primary and Ar^+^ as sputtering ion guns. The cross-section images are stretched along the z-axis for clarity and the scale bar represents the horizontal spacing. **e** Depth profile of (Co+Fe)/(La+Sr) for the −0.1 V, −0.35 V, and −0.43 V LSCF-SDC electrodes.

To connect the surface to the sub-surface chemical changes, we utilized time of flight secondary ions mass spectrometry (ToF-SIMS or SIMS) with depth profiling of different elements. Figure 3 shows the SIMS analysis and reconstructed cross-section images of LSCF-SDC cathode electrodes. Argon sputter gun was used for the 3D analysis of different cells along with Bi_3_^++^ as the primary ion gun (see Fig. S8-S13 and method section for more details). Here we chose cathode electrodes of −0.1 V, −0.35 V, and −0.43 V for 3D analysis because of the activation and degradation behavior of LSCF-SDC polarized at lower and higher potentials. Stark differences in A-site and B-site cation signals as a function of sputtering depth (Fig. 3a-c) can be observed between the −0.1 V, −0.35 V, and −0.43 V cathodes. Figure 3d shows cross-section maps (*x-z* view) of Sr^+^ count for these electrodes. As can be seen in Fig. 3a and Fig. 3d, the −0.1 V electrode is heavily Sr-rich near its surface, with a gradual decrease in Sr signal as the sputtering progresses into the bulk of the LSCF-SDC electrode. On the other hand, an opposite trend in the Sr signal can be observed in −0.35 V and −0.43 V electrodes, with a low Sr signal at the surface that gradually increases with sputtering depth into the bulk. As expected, the abundance or scarcity of Sr on the surface coincides with an opposite trend in the Co and Fe signals. This finding from SIMS is consistent with the XPS results shown in Fig. 2d, pointing to an increase in B-site cations with increasing voltage.

Figure 3e shows the B/A ratio semiquantitatively for −0.1 V, −0.35 V, and −0.43 V LSCF-SDC electrodes as a function of sputtering depth (see Methods section for more details). LSCF-SDC with an overpotential of −0.1 V shows a monotonic increase in B/A, mainly due to the strong segregation of Sr at the surface of this electrode. On the other hand, the depth profile of B/A for the −0.35 and −0.43 V electrodes can be divided into three different zones. Zone I is the region where we observe a sharp drop in the B/A ratio as a function of sputtering depth (within about 5 nm of the surface). Zone II is the region in which we observe a drop in the B/A ratio with a smaller slope compared to the surface but in a longer range. This points to the depletion of B-site cations in this sub-surface region ( ~ 5-30 nm). Zone III is the region where the B/A depth profile shows an increase with a trajectory that reaches the bulk value of B/A.

**Fig. 4:**
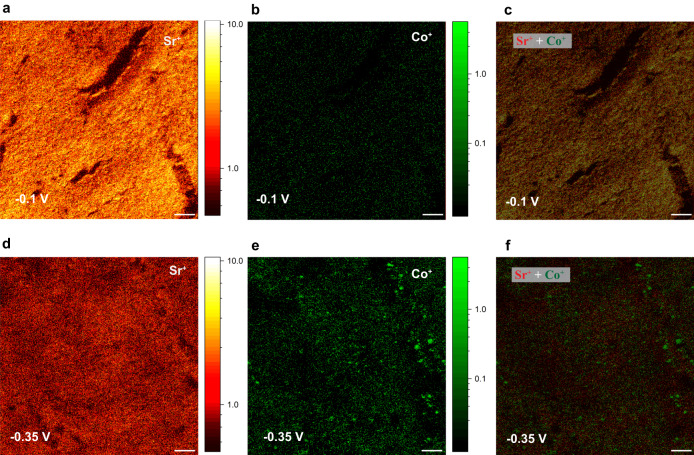
High-resolution 2D SIMS images in unbunched mode. **a**–**c** show the Sr^+^, Co^+^, and overlay of Sr^+^ and Co^+^ for −0.1 V electrode. **d**–**f** show the Sr^+^, Co^+^, and overlay of Sr^+^ and Co^+^ for −0.35 V electrode. The scale bars show 10 µm and the color bar represents the count per pixel on each image.

To further resolve the Co and Fe distribution, we employ high spatial resolution 2D SIMS maps. Figure 4 shows SIMS 2D elemental maps of Sr^+^ and Co^+^ ions for −0.1 V and −0.35 V electrodes. As can be seen, the Sr^+^ surface map of the −0.1 V electrode has a higher intensity compared to the −0.35 V electrode. While Co^+^ shows a significantly higher intensity in the -0.35 V electrode compared to -0.1 V. This is consistent with the depth profile results shown in Fig. 3. In addition, we observe that the Co^+^ surface map of the -0.1 V electrode is mainly uniform while increasing the overpotential to -0.35 V leads to a clear formation of micrometer size Co-rich exsolved particles on the surface.

### Formation of a Ruddlesden–Popper phase

**Fig. 5:**
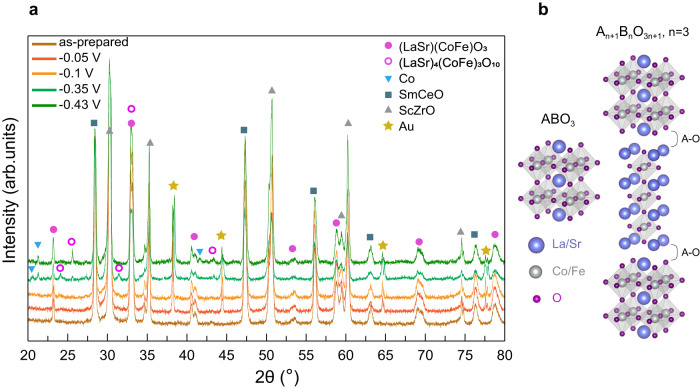
XRD pattern of LSCF-SDC electrode upon different applied cathodic potentials. **a** Ex-situ X-ray diffraction pattern is obtained after quenching the cells from 800 °C to room temperature at each applied overpotential. The diffraction pattern is measured on the cathode electrode. **b** Schematic of the crystal structures of the P phase and RP3 phase (VESTA software is used for visualization)^66^.

X-ray diffraction was used for analyzing the structure of LSCF-SDC electrodes polarized at different voltages. Figure 5a shows the diffraction pattern and the assigned peaks for different components. While the LSCF-SDC electrodes with cathodic overpotentials of −0.05 V and −0.1 V show an identical structure to the as-prepared electrode, the -0.35 V and -0.43 V electrodes show additional peaks that do not exist in the as-prepared, -0.05 V, and −0.1 V electrodes. These additional peaks correspond to the formation of A_4_B_3_O_10_ (RP3, n = 3 in A_n+1_B_n_O_3n+1_) or Ruddlesden-Popper (RP3) perovskite in LSCF-SDC electrodes polarized at higher overpotentials. Figure 5b shows the crystal structure of the perovskite (P) phase and the RP3 phase, the latter with three layers of perovskite separated by an A-O rock-salt layer along the c-axis^45^. The RP3 phase has a tetragonal structure (I4/mmm space group, lattice parameters of a = b = 3.84 Å and c = 27.87 Å), and the P has a Rhombohedral structure (R-3c space group, lattice parameters of a = b = 5.45 Å, c = 13.19 Å). Due to the polycrystalline and porous nature of these samples, some RP3 peaks are not consistently present when comparing the X-ray diffraction patterns for −0.35 V and −0.43 V electrodes. This is possibly due to the preferred orientation of RP3 phase formation in each sample. (See Fig. S14 for more information). In addition to the formation of RP3 phase, several peaks which were assigned to cobalt metal, appear for the −0.35 V and −0.43 V electrodes.

### Electrochemical polarization of LSCF

In addition to the observed electrochemical improvement in LSCF-SDC due to applied cathodic polarization, we have utilized this method in LSCF electrodes (without SDC mixed in composite). We have used symmetric cells made of LSCF electrodes on both sides of the ScSZ electrolyte. This experiment helps to resolve whether the SDC has any significant role in the activation of the oxygen electrode under cathodic polarization. The polarization resistance and current density of LSCF at cell voltages of 0.2 V and 0.6 V are shown in Fig. S15. As can be seen at 0.6 V cell voltage, LSCF current density is increasing and polarization resistance is decreasing which indicates activation, whereas the opposite trend is observed when polarizing the LSCF at 0.2 V cell voltage, showing degradation. By applying a cell voltage of 0.6 V and 0.2 V, an overpotential of −0.33 V and −0.1 V have been applied on the cathodic electrodes respectively. To understand the mechanism behind this activation, we perform surface and structural characterization on the cathodic electrode of LSCF. Fig. S16 shows the X-ray diffraction pattern of −0.33 V LSCF electrode and as-prepared LSCF. Additional peaks formed in the −0.33 V LSCF electrode diffraction pattern are assigned to A_4_B_3_O_10_ (RP3, *n* = 3 in A_n+1_B_n_O_3n+1_) or Ruddlesden-Popper (RP3) perovskite. In addition, several peaks corresponding to cobalt metal appear for the −0.33 V LSCF electrode. These are consistent with our findings of the cathodically polarized and electrochemically improved LSCF-SDC composite electrode. In addition, XPS analysis shows that the Sr surface and lattice components decrease and increase, respectively, and Co and Fe enrich on the surface of the −0.33 V LSCF electrode compared to the as-prepared LSCF (Table S1). Thus the surface chemistry evolution of the cathodically polarized LSCF is similar to that in the LSCF-SDC composite electrode. This concludes that SDC has no detectable role in this cathodic activation behavior of LSCF.

### Electrochemical performance of fuel cell

**Fig. 6:**
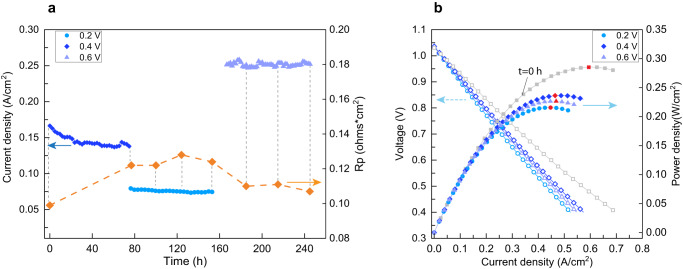
Electrochemical performance of fuel cell upon different applied voltages. **a** Current density as a function of time at different applied voltages and oxygen electrode polarization resistance at OCV at t = 0 h and after applying each voltage for 80 h. **b** j-V-P curve of the fuel cell after applying each voltage and at the beginning of the experiment at t = 0 h. The maximum power density is indicated by a red symbol on each curve.

To explore the applicability of the applied cathodic polarization method, the electrochemical stability of fuel cells at lower (0.2 V, 0.4 V) and higher (0.6 V) voltage regimes were evaluated. We fabricated fuel cells with NCS composite (NiO | Sm_0.2_Ce_0.8_O_1.9_, 70 | 30 ratios by weight) as fuel electrodes and LSCF-SDC composite as oxygen electrodes on ScSZ electrolyte. The electrochemical tests were carried out at 800 °C with 3% H_2_ and 3% N_2_ as fuel and ambient air as the oxidant under fuel cell voltages of 0.2 V, 0.4 V, and 0.6 V. As can be seen in Fig. 6, after applying 0.2 V and 0.4 V, the polarization resistance of the oxygen electrode (measured by the reference electrode) increases and the current density decreases, indicating degradation. On the other hand, by increasing the cell voltage to 0.6 V, the polarization resistance of the oxygen electrode decreases, and current density shows stability until the end of the measurement period. Thus, increasing the voltage improves the electrochemical performance of the fuel cell.

The current density-potential-power (j-V-P) curves are measured at the beginning of the experiment (t = 0 h) and after applying each cell voltage for 80 hours. When comparing the power densities, we observe that by applying voltages of 0.2 V and 0.4 V, the maximum power density (P_max_, indicated by a red symbol on each curve) decreases compared to the initial P_max_, however, the P_max_ increases after applying 0.6 V, showing activation. It is worth mentioning that the P_max_ after 0.6 V is smaller than the P_max_ after 0.4 V and the initial P_max_. The reason behind this drop in P_max_ is the degradation of the fuel electrode polarization resistance. Fig. S17 shows the polarization resistance of the NCS fuel electrode measured at OCV. The *Rp* of the NCS electrode increases (degradation) by applying 0.2 V and 0.4 V and decreases (activation) by applying 0.6 V. Even though we observe activation of NCS *Rp* by increasing the voltage from 0.2 V to 0.6 V, the *Rp* at t = 240 h is still larger than t = 0 h and t = 80 h and some degradation of the NCS fuel electrode remains. Therefore, we conclude that the cathodic activation of LSCF-SDC is valid not only in the symmetric cells that we have reported above but also in full solid oxide fuel cells.

## Discussion

Reconciling the results described above, we observe a relation between the improved activity and stability in LSCF-SDC electrodes under large cathodic overpotentials, and the suppression of Sr segregation, Co exsolution at the surface, and RP3 phase formation. Here the suppression of the Sr surface component on the LSCF-SDC electrodes with electrochemical overpotentials of −0.35 V and −0.43 V coincides with the improved oxygen reduction reaction rate shown in Figs. 1 and 2. It has been studied extensively in the literature that Sr segregation, termination or phase precipitation at the surface can cause degradation of electrochemical activity in perovskite oxygen electrodes^22,27,28,46,47^. Hence, suppression of the Sr-rich insulating phase at the surface of -0.35 V and -0.43 V electrodes leads to more available active sites for oxygen reduction reactions. In contrast, segregated Sr species in electrodes with smaller overpotentials than -0.35 V, block the active sites for oxygen reduction reactions. Subsequently, degradation occurs, which is observed by an increase in the *Rp* values and a decrease in current densities. In addition to the suppression of Sr-surface content in −0.35 V and −0.43 V electrodes, the formation of exsolved particles of Co shown in Fig. 4 is another outcome of phase decomposition at larger polarizations.

The RP phase can also contribute to the improvement of the surface ORR activity. Previously we showed that the presence of the RP phase in a composite of RP1 (*n* = 1) and P phases of (La_0.5_Sr_0.5_)_2_CoO_4_ (LSC_214_) and La_0.8_Sr_0.2_CoO_3_ (LSC_113_) thin films decreases the precipitation of Sr to the surface compared to the LSC_113_ thin film consisting of only the P phase^48^. So, a similar mechanism could be associated with the suppression of Sr segregated species on the surface of LSCF-SDC electrodes with overpotentials greater than -0.35 V. One can also consider the role of oxygen interstitials of RP3 in improving activity and stability^49,50^. Because of the presence of two A-O rock-salt layers in the RP3 phase, it can assist with oxygen incorporation^51,52^.

To elucidate the underlying mechanism behind the RP3 phase formation, Co exsolution, and suppression of Sr-surface, we first consider the decomposition of the LSCF-SDC electrodes under the cathodic polarization^53^. By increasing the electrochemical polarization, an overpotential of -0.35 V and -0.43 V were applied to the LSCF-SDC cathodes, respectively. The oxygen partial pressure on the cathode as a function of over potential is derived based on the Nernst equation:

1 $${P}_{{O}_{2}}={P}_{{O}_{2,gas}}exp\left(-\eta \frac{4F}{RT}\right)$$

where P_O2,*gas*_ is the atmospheric oxygen partial pressure, ɳ is the overpotential, $F$ is Faraday’s constant, $R$ is the universal gas constant, and $T$ is the absolute temperature. Therefore, −0.43 V and −0.35 V overpotentials result in an oxygen partial pressure of ~10^−9 ^bar and ~10^−8 ^bar, respectively. Fig. S18 shows the calculated oxygen partial pressures for all the overpotentials. Based on coulometric titration, the decomposition of La_1-x_Sr_x_Co_1-y_Fe_y_O_3_, y = 0.2, perovskite starts at ~10^-6 ^bar at 800 °C which precipitates the RP phase^53^. Under this reducing condition, Co cation simultaneously leaves the parent perovskite lattice and precipitates to the surface where the formation of exsolved Co particles takes place^53,54^. Exsolution of transition metals in perovskite oxides has been shown to take place upon exposure to a reducing environment, typical H_2_ gas at elevated temperatures, or reducing cathodic potentials in the presence of H_2_O or CO_2_, where the transition metal cation reduces, leaves the parent perovskite and precipitates as metal nanoparticles at the surface^55–61^. However, it is intriguing to observe exsolved Co metal particles in ambient air on the LSCF-SDC cathodic electrode. In this case, the exsolution of Co particles at the surface of LSCF-SDC electrodes with overpotentials of −0.43 V and −0.35 V can be explained by the effective oxygen partial pressure being very low as noted above, based on the Nernst relation (Eq. 1). The presence of Co exsolved particles on the surface, in turn, can increase the oxygen reduction reaction rate. Prior research has shown that the catalytic activity of Co exsolved particles increases the rate of oxygen reduction reactions at the surface of the perovskite oxides by facilitating the electron transfer and oxygen dissociation steps^62–65^.

As discussed before, −0.1 V, −0.35 V, and −0.43 V electrodes show a drastically different 1D SIMS depth profile of B/A ratios (Fig. 3e). A drop in the B/A ratio of -0.35 V and -0.43 V electrodes, indicates a B-site deficient zone in the sub-surface region (~5−30 nm). On the other hand, the −0.1 V electrode shows an opposite trend in which the B/A ratio increases continuously up to 10-15 nm below the surface and plateaus after that depth. That is also consistent with the lack of Co exsolved particles on the surface of the -0.1 V electrode. We conclude that the formation of B-site deficient A_4_B_3_O_10_, RP3 phase takes place in the sub-surface of LSCF-SDC within the Co and Fe-deficient region shown in SIMS profiles.

By comparing the electrochemical performance of LSCF (Fig. S15) and LSCF-SDC (Fig. 1), surface chemistry evolution (Tabel. S1 for LSCF and Fig. 2 for LSCF-SDC), and X-ray diffraction patterns (Fig. S16 for LSCF and Fig. 5 for LSCF-SDC), we conclude that the LSCF electrode is activated and becomes stable under similar cathodic conditions as that of the LSCF-SDC electrode. This experiment confirms that LSCF alone is dominantly activated under cathodic polarization, and the SDC phase in the composite does not play a significant role in such activation.

In addition, based on the electrochemical performance of the full solid oxide fuel cell presented in Fig. 6 and the electrochemical performance of symmetric cells presented in Fig. 1, the activation of the LSCF-SDC oxygen electrode at moderate cathodic overpotentials is not limited to symmetric electrodes in ambient air but is also applicable under realistic fuel cell conditions. The activation of the LSCF-SDC oxygen electrode is accompanied by a relative improvement in the maximum power density and stability in the current density of the fuel cell. The full cell tests were carried out in SOFC mode where the oxygen electrode was cathodically polarized and is the focus of this study. The reason is that the cathodic electrode shows a larger extent of chemical and electrochemical improvement compared to the anodic electrode. The dominant changes in the cathodic electrode can be seen from how much the *Rp* changes as shown in Fig. 1c and Fig. 1d, and the near-surface chemistry evolution in Fig. 2 and Fig. S6. In addition, the formation of the RP3 phase and exsolution of Co only happen on the cathodic electrode.

In conclusion, we assessed the oxygen reduction activity and stability as a function of electrochemical polarization in porous LSCF-SDC electrodes in a symmetric cell configuration. Our results show that the surface chemical stability and electrochemical performance of LSCF-SDC electrodes have been improved over a long period of testing by polarizing the cells above 0.6 V. The increased cathodic polarization decomposes the perovskite phase which facilitates the formation of the RP3 phase that takes place in the sub-surface of the LSCF-SDC electrode. We show that the superior chemical and electrochemical stability of RP3/P mixed composite is due to the suppression of Sr surface species and promotion of Co and Fe precipitation to the surface that results in the formation of Co exsolved particles. We further show that the precipitation of B-site cations on the surface leads to the formation of a B-site depleted sub-surface region which is associated with the RP3 formation zone. Similar experiments on oxygen electrodes made of LSCF confirm that the electrode activation under cathodic overpotentials is arising predominantly from LSCF, and not from SDC. We have also confirmed the applicability of this approach and the aforementioned findings on the cathodic activation of LSCF-SDC under realistic fuel cell conditions. This work introduces a viable approach for improving the stability and activity of LSCF-SDC oxygen electrodes in both solid oxide fuel and electrolysis cells.

## Methods

### Sample preparation

Symmetric button cell samples were prepared by OxEon Energy using tape casting and sintering for the ScSZ electrolyte support, followed by screen printing and sintering for the SDC barrier layers and LSCF-SDC electrodes on each side. The LSCF-SDC composite layer is 50:50 by weight. The electrolyte support was fabricated to be approximately 20 mm in diameter and 0.23 mm thick. The electrodes were printed with a 6 × 6 mm active electrode and a 2 × 1 mm reference electrode positioned 1.1 mm away from the active electrode. The distance between the active and reference electrode should be three times larger than the thickness of the electrolyte because only in this equipotential region the reference electrode can be used correctly.

Fuel cell samples were prepared by OxEon Energy using tape casting and sintering for the ScSZ electrolyte support, followed by screen printing, and sintering for the SDC barrier layers and LSCF-SDC electrodes as oxygen electrodes and NCS (NiO | Sm_0.2_Ce_0.8_O_1.9_, 70 | 30 ratios by weight) as fuel electrodes. The electrolyte support was fabricated to be approximately 36 mm in diameter and 0.21 mm thick. The electrodes were printed with a 1.41 × 1.41 cm active electrode (2.0 cm^2^ active area) and a 3 × 3 mm reference electrode positioned 2 mm away from the active electrode. The distance between the active and reference electrode should be three times larger than the thickness of the electrolyte because only in this equipotential region the reference electrode can be used correctly.

### XPS measurements and analysis

PHI Versaprobe II XPS is used to perform ex-situ X-ray photoelectron spectroscopy on the surface of the LSCF-SDC porous electrode with a mono-energetic Al Kα X-rays source. C60 cluster-ion gun is used to neutralize the surface charges and allow the emission of photoelectrons. CasaXPS software is used to fit the XPS core level spectra and for quantitative analysis of La *3d*, Co *2p*, Co *3p*, Fe *3p*, and Sr *3d*. XPS measurements are performed at room temperature and a base pressure of 10^−10 ^Torr. XPS peaks are calibrated with respect to the C *1* *s* peak.

### EIS

Electrochemical impedance spectroscopy measurement was carried out by using Parstat 4000 A potentiostat. The impedance spectra at the open circuit voltage in the frequency range 100 kHz–3 mHz with an a.c. amplitude of 10 mV for symmetric cells of LSCF-SDC is measured for two-electrode and three-electrode configurations. The chronoamperometry measurement is performed for polarization application on the active electrodes with two-electrode configuration. The gold paste is used to attach the gold mesh to the cells and gold wires are attached to the gold mesh on both sides of the cell to apply cathodic and anodic polarization to each electrode. The gold wires are connected to the working lead and the counter lead of the potentiostat during the chronoamperometry measurement and to measure the EIS at open circuit voltage using the reference electrode, the reference lead of the potentiostat is connected to the reference electrode. EIS results are fitted and analyzed by using ZView software. There was no iR-compensation performed.

### XRD

The PANalytical XPert Pro X-ray diffraction equipped with a Cu target is used for the structural characterization of LSCF-SDC symmetric cells. A default configuration of this instrument in Bragg-Brentano geometry is used for 2theta-that measurement in the z-axis and between 20 degrees and 80 degrees. Highscore software is used for data analysis and peak refinement.

### ToF-SIMS measurements

ToF-SIMS experiments were performed using a PHI nanoTOF II instrument equipped with a bismuth liquid metal ion gun (LMIG), an Ar^+^ sputtering gun, and an electron neutralization gun for charge compensation. A burst alignment imaging mode was used to provide high lateral resolution and chemical resolution simultaneously, where Bi_3_^++^ was employed as the primary ion and Ar^+^ was employed as the sputtering source. Typical measurement conditions were ∼0.5 nA Bi_3_^++^ at 30 keV into a 50 × 50 μm^2^ area. The LMIG current was measured using a faraday cup within the LMIG column. This current corresponds to ~7 × 10^12^ ion/cm^2^ for 2D images and ~3 × 10^12^ per cycle of 3D depth profile scan. To avoid the edge effect during the mass sputtering a sputter area of 500 × 500 μm^2^ was selected. The sputter beam was Ar^+^ at 100 nA at 4 keV. Detection of positive ions was appropriate because of the high secondary ion yields for Sr^+^, La^+^, Co^+,^ and Fe^+^. 3D profiles are acquired using data collection over 60 cycles, each cycle is collected after 5 seconds of sputtering with a 2-second delay for charge compensation after sputtering. Each cycle of a 3D image has 256 × 256 pixel^2^ while the 512 × 512 pixel^2^ setting is used for 2D data collection to increase the lateral resolution. Both bismuth and argon ion columns are oriented at 60° with respect to the sample surface normal. The analysis chamber pressure is maintained below ~2 × 10^−9^ mbar to avoid contamination of the surfaces to be analyzed.

The semi-quantitative A/B ratios presented in Fig. 3e are obtained by using the compositional values extracted from the cross-section XPS of the as-prepared electrode. A thick LSCF-SDC pellet sample was prepared and cleaved for cross-section XPS. Subsequently, 1D SIMS depth profiles were normalized by constant values for each element in a manner that the average of the last five data points for a given SIMS depth profile (representing the bulk value of the element under discussion) is equal to the composition value extracted from cross-section XPS. This normalization process is done to account for different secondary ions yield of various A- and B-site cations in the SIMS experiment. We must emphasize that B/A values represented in Fig. 3e are semi-quantitative values used here for the purpose of comparing chemical changes on the surface *versus* sub-surface regions and should not be interpreted as quantitative compositional values.

### Supplementary information

Supplementary Information

Peer Review File

## Data Availability

The datasets generated and/or analyzed during the current study are available from the corresponding authors upon request.
