# Supplementary material for: Improvement of oxygen reduction activity and stability on a perovskite oxide surface by electrochemical potential
Source: Nat Commun. 2023 Nov 8;14:7203. doi: 10.1038/s41467-023-42462-5 (PMC10632449; doi:10.1038/s41467-023-42462-5)
Supplement: Supplementary file 1 — Supplementary Information [file 41467_2023_42462_MOESM1_ESM.pdf]

Supplementary Information

Improvement of Oxygen Reduction Activity and Stability on a Perovskite Oxide Surface by Electrochemical Potential

*Sanaz Koohfar[1,2], Masoud Ghasemi[3,4], Tyler Hafen[5], Georgios Dimitrakopoulos[6], Dongha Kim[6], Jenna Pike[5], Singaravelu Elangovan[5], Enrique D. Gomez[3,4], Bilge Yildiz[1,2,6]**

[1] Department of Nuclear Science and Engineering, Massachusetts Institute of Technology, 77 Massachusetts Avenue, Cambridge, Massachusetts, 02139, USA.

[2] Laboratory for Electrochemical Interfaces, Massachusetts Institute of Technology, 77 Massachusetts Avenue, Cambridge, Massachusetts, 02139, USA.

[3] Department of Chemical Engineering, The Pennsylvania State University, University Park, Pennsylvania, 16802, USA

[4] Department of Materials Science and Engineering, The Pennsylvania State University, University Park, Pennsylvania, 16802, USA

[5] OxEon Energy, LLC, North Salt Lake, Utah, 84054, USA

[6] Department of Material Science and Engineering, Massachusetts Institute of Technology, 77 Massachusetts Avenue, Cambridge, Massachusetts, 02139, USA.

*To whom correspondence should be addressed. Email: byildiz@mit.edu

Supplementary Information

Validation of reference electrode measurement

We compare the polarization resistance measured by two and three electrodes configurations to validate the correct placement of the reference electrode. Fig. S1 shows the polarization resistance measured by the three electrodes (*Rp1* and *Rp2*) and two electrodes (*Rp* total) configurations. The sum of *Rp1* and *Rp2* (0.37 Ωcm^2 + 0.32 Ωcm^2=0.69 Ωcm^2) equals *Rp* total (0.69 Ωcm^2) which validates the required criteria in placement of the reference electrode.

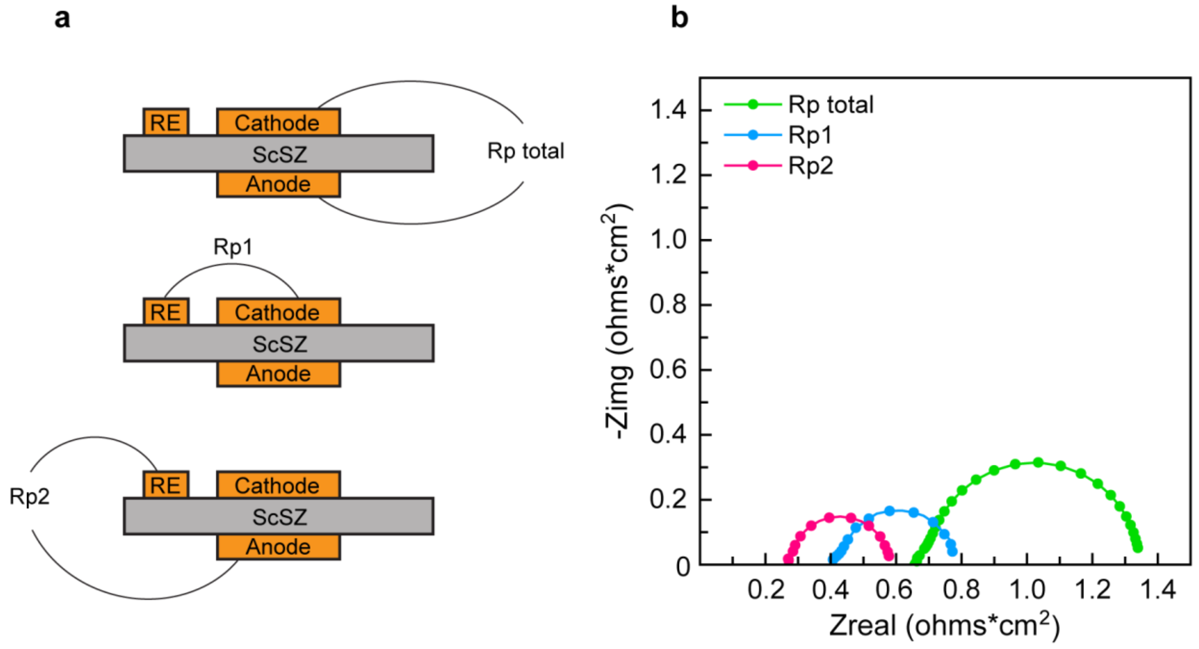

Fig. S1. Polarization resistance measured by two and three electrodes configurations. a, Schematic of the two electrodes (top) and three electrodes (middle and bottom) measurement. **b,** *Rp* total is measured across the cell by using the two electrodes configuration. *Rp1* and *Rp2* are measured by three electrode configurations representing the cathodic and anodic electrodes respectively.

Calculation of polarization resistance

Fig. S2 shows the impedance semicircle for the symmetric cell of LSCF-SDC on ScSZ electrolyte at open circuit voltage. The inset shows the equivalent circuit model to fit the ESI result[1, 2]. The equivalent circuit model consists of one R//C for high frequencies and one R//CPE for lower frequencies. The lower frequency arc represents diffusion processes which include adsorption and diffusion of oxygen and oxygen exchange on the surface of LSCF-SDC and at the interface of LSCF-SDC and ScSZ. A higher frequency arc is associated with charge transfer which includes oxide ion diffusion in the bulk and incorporation of oxygen ions. Polarization resistance, Rp, is the sum of these two arcs at Zimg=0.

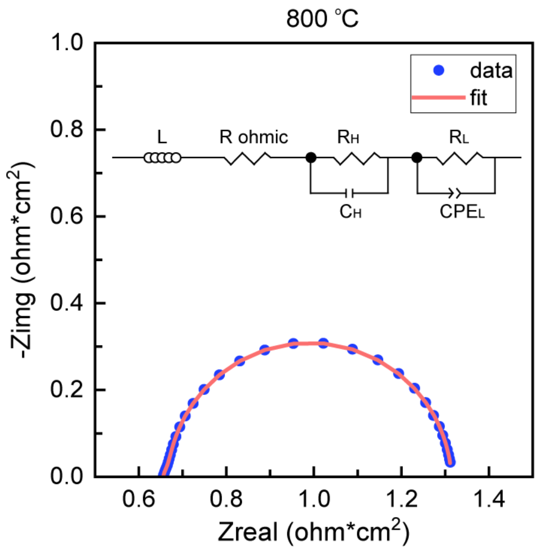

Fig. S2. Analysis of impedance semicircle of LSCF-SDC symmetric cell on ScSZ electrolyte at 800 °C in ambient air. The equivalent circuit model is used to fit the data which consists of two arcs at lower and higher frequencies.

Current density and polarization resistance relation

Fig. S3 shows the comparison between the *Rp* and current densities for 0.6 V and 0.8 V. At 0.8 V, the cell continues to activate during the 80 hours of operation and an increase in the current density is consistent with a decrease in polarization resistance. The arrows in Fig. S3 b indicate the increase in current density with respect to the initial value at time 0 h.

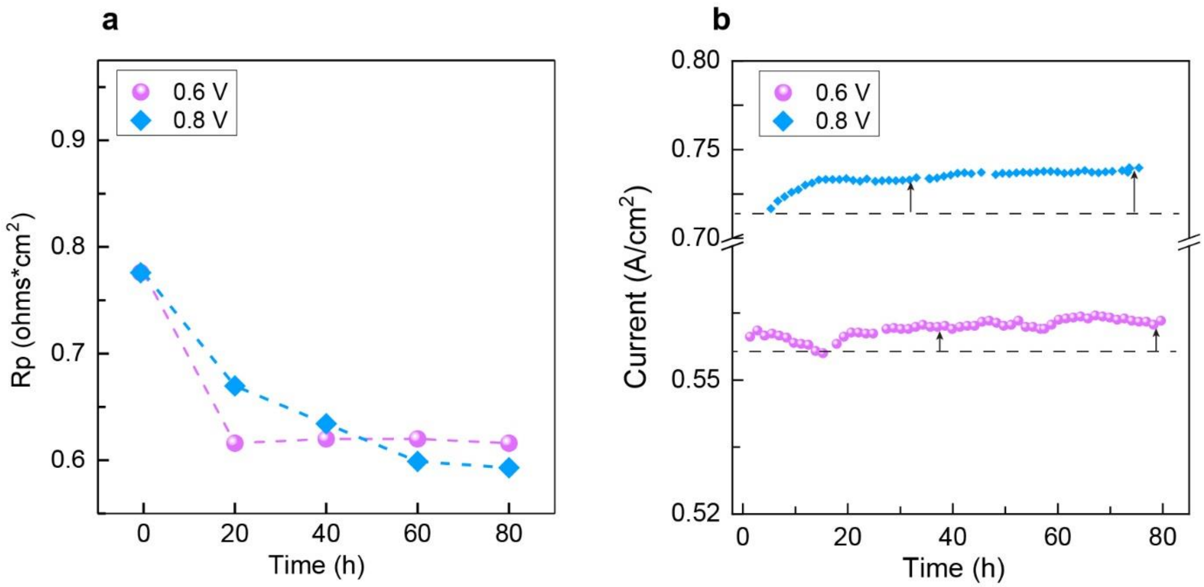

Fig. S3. Comparison of polarization resistance and current density. a, Polarization resitance measured at OCV for 0.6 V and 0.8 v cells. **b,** Current denisty of 0.6 V and 0.8 V. The arrows indicate the increase in current density with respect to their initial values.

Regarding the 0.6 V cell, the current density between the 60 hours and 75 hours of measurement has a slight increase and plateaus after 75 hours and the current density at 52 hours and 78 hours has similar values. Because there is a smaller increase in current density at 0.6 V compared to 0.8 V (by comparing the arrows in Fig. S3 b), the *Rp* of 0.6 V cell shows smaller activation when compared to the *Rp* of 0.8 V cell. Therefore, the current density and *Rp* behave similarly for each cell but behave differently when comparing the 0.6 V and 0.8 V cells.

Fig. S3 shows the comparison between the *Rp* and current densities for 0.6 V and 0.8 V. At 0.8 V, the cell continues to activate during the 80 hours of operation and an increase in the current density is consistent with a decrease in polarization resistance. The arrows in Fig. S3 b indicate the increase in current density with respect to the initial value at time 0 h.

X-ray photoelectron spectroscopy measurement of cathode

Fig. S4 and Fig. S5 show Co *2p*, Co *3p*, Fe *3p*, and La *3d* core level spectra and their deconvolution. CasaXPS software was used to fit the XPS spectra. An asymmetric Lorentzian line shape with a fixed Gaussian width is used to fit the data. La *3d* spectra are deconvoluted to perovskite lattice-bound at lower binding energy and the peak at higher binding energy is the contribution of surface-bound La. The plasmons peak of La *3d5/2* and La *3d3/2* are shown. Co $2p_{3/2}$ photoelectron core level shows two different oxidation states of Co^{2+} and Co^{3+}.

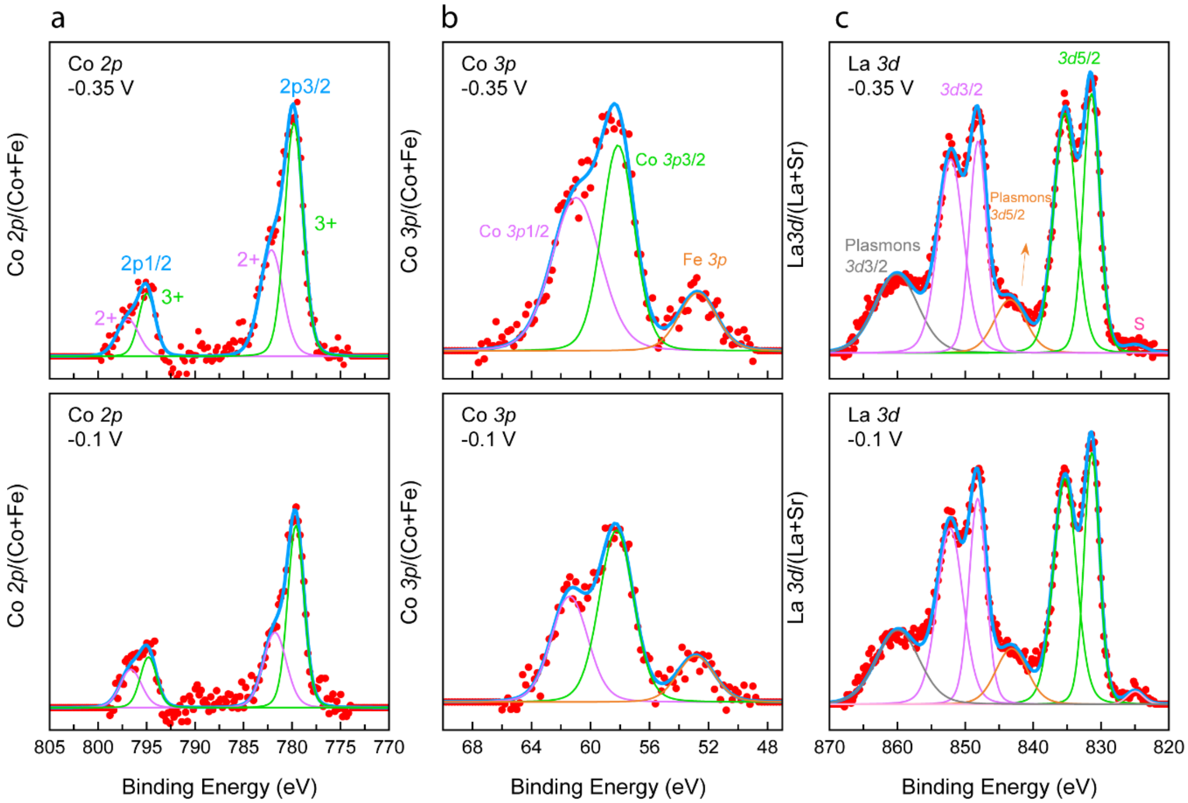

Fig. S4. XPS core level spectra of the cathodic electrode. a, Co *2p* normalized to the B-site cations **b,** Co *3p* and Fe *3p* normalized to the B-site cations **c,** La *3d* normalized to the A-site cations for cathode electrode of 0.6 V cell on the top and cathode electrode of 0.2 V cell on the bottom. The overpotential applied on the cathode is indicated in the figures. Data is the red points, and the fit is the blue line.

X-ray photoelectron spectroscopy measurement of anode

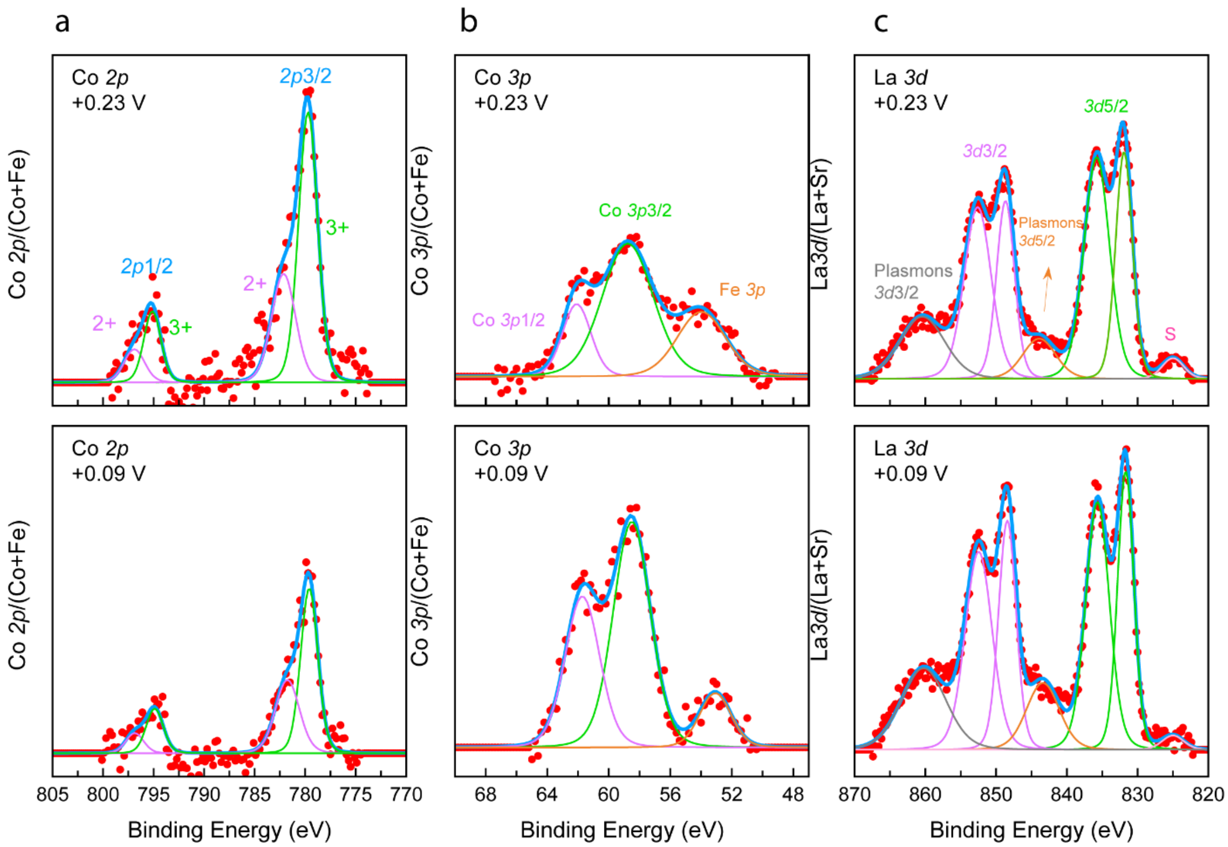

Fig. S5. XPS core level spectra of the anodic electrode. **a,** Co *2p* normalized to the B-site cations **b,** Co *3p* and Fe *3p* normalized to the B-site cations **c,** La 3d normalized to the A-site cations for an anode electrode of 0.6 V cell on the top and anode electrode of 0.2 V cell on the bottom. The overpotential applied on the anode is indicated in the figures. Data is the red points, and the fit is the blue line.

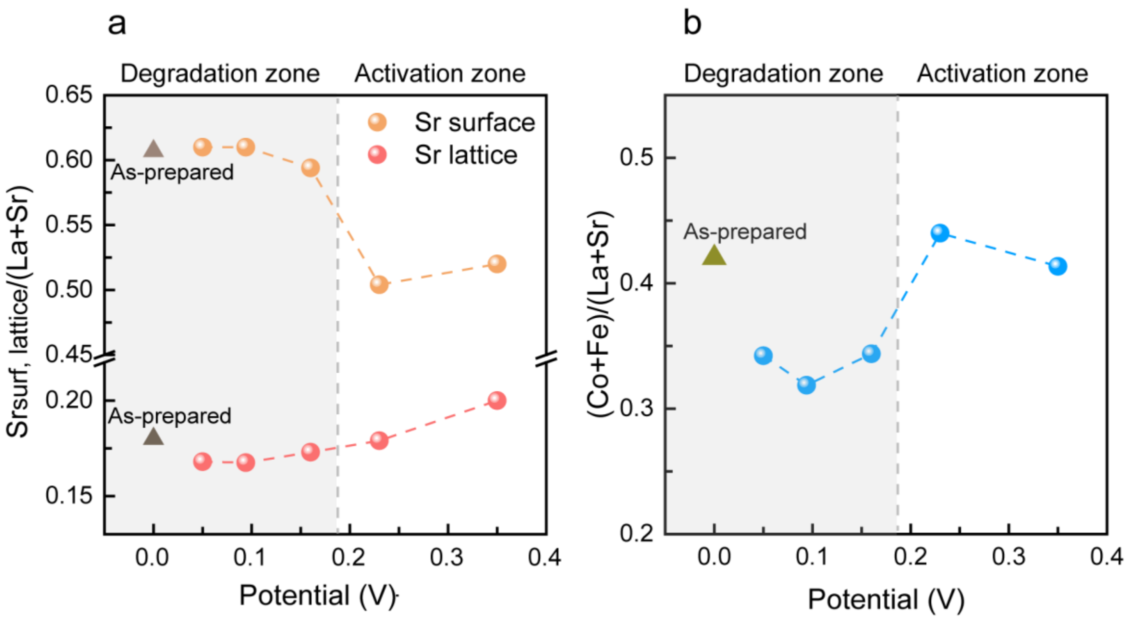

Fig. S6. Surface chemistry evolution with anodic overpotential a, Surface and lattice concentration ration of Sr normalized to total A-site (La and Sr) on the surface of the anode. **b**, The ratio of B-site cations (Co and Fe) to A-site cations (La and Sr) on the surface of the anode. The triangles show the respective quantity measured on as-prepared cells. The x-axis of each data point represents the overpotential measured by using a reference electrode and the grey area indicates the deactivation or degradation zone.

We further investigate the structural and chemical changes of anodic electrodes by performing X-ray diffraction and XPS, respectively. Fig. S7 shows the X-ray diffraction pattern of anodic and cathodic electrodes of 0.8 V cell. The anodic electrode does not show additional peaks related to the secondary phase compared to the cathodic electrode which is expected because the oxidizing conditions on anode electrodes do not promote reduction and phase decomposition of P phases. Despite the lack of secondary phases in anodic electrodes, we observe a slight improvement in the electrochemical activation as shown in Fig. 1c which is consistent with the suppression of Sr non-lattice and an increase in the B-site cations ratios as can be seen in Fig. S6. We thus associate the observed behavior of the anodic electrode with a mechanism different from the mechanisms observed on cathodic electrodes. Characterization and identification of this mechanism are subject to further studies that are outside the scope of the current work.

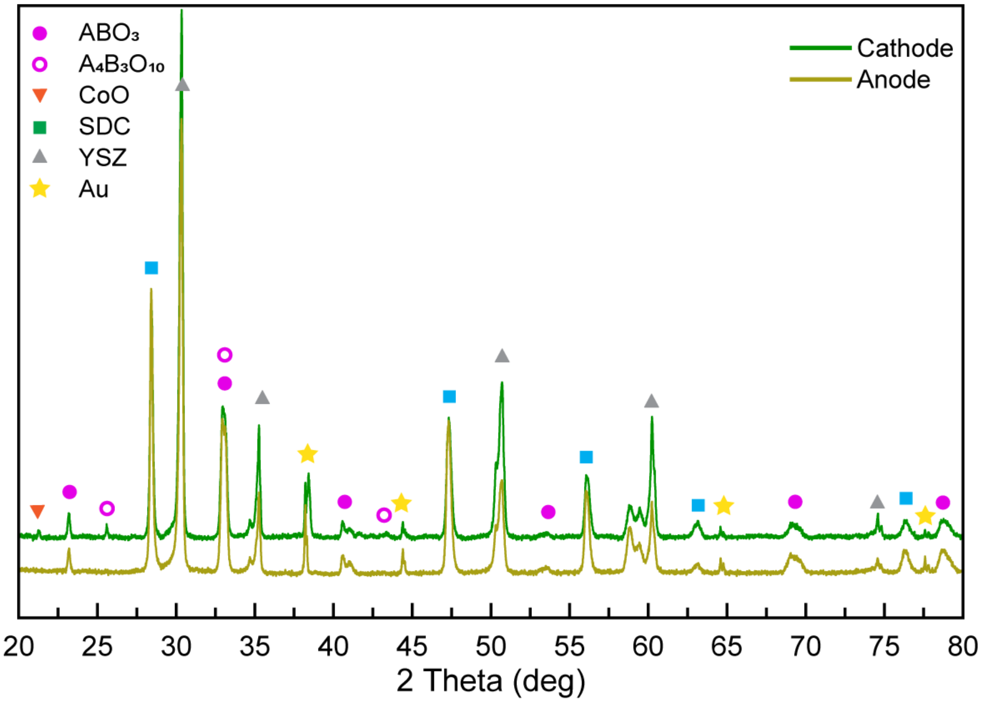

Fig. S7. X-ray diffraction pattern of the anode and cathode electrode of 0.8 V cell. No additional peaks are forming on the anode electrode

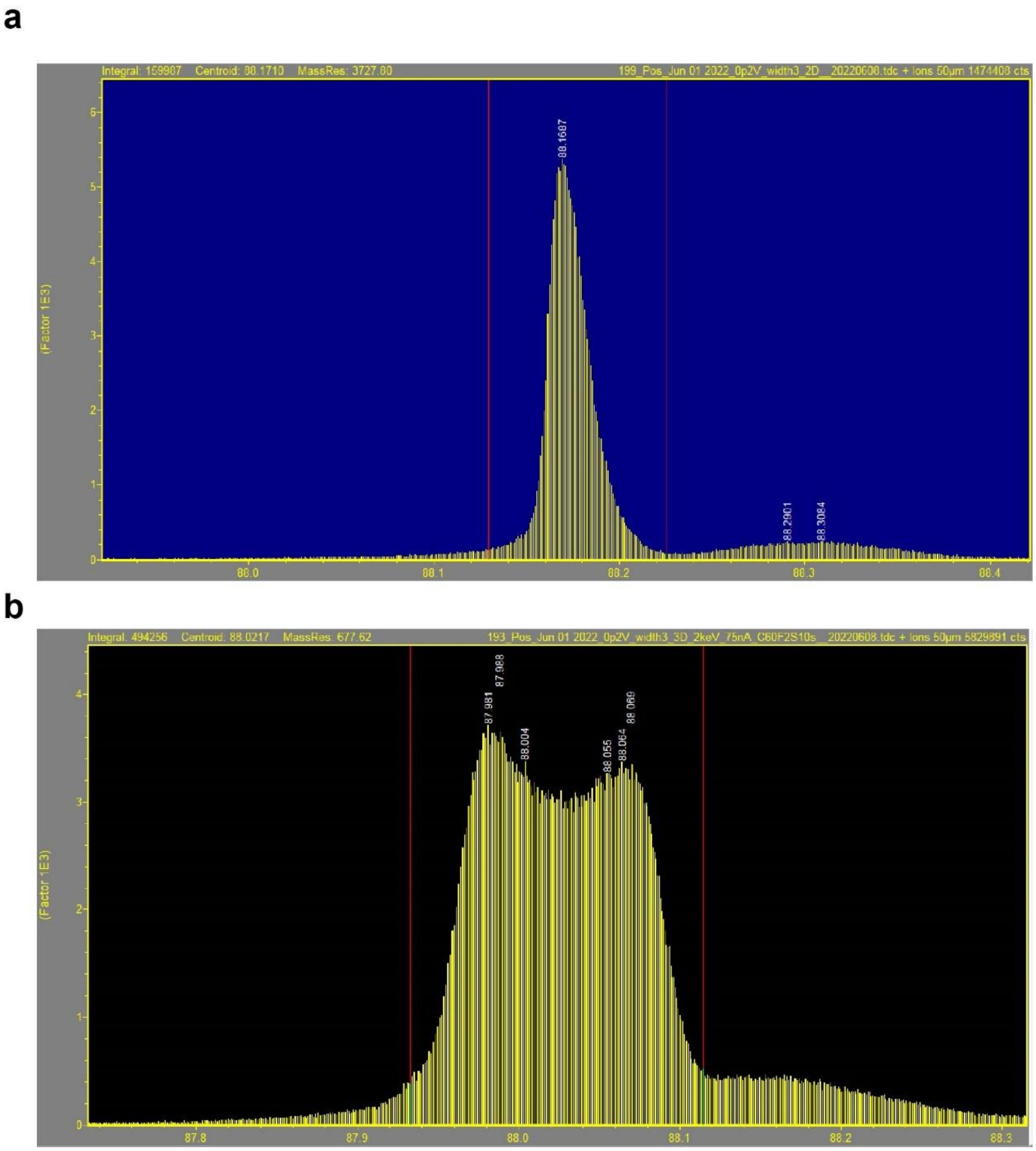

Fig. S8. Bunched versus unbunched Sr$^+$ peak. in **a**, bunched, and **b**, unbunched modes. 6 V and 0 V buncher voltages were used in bunched and unpunched mode, respectively.

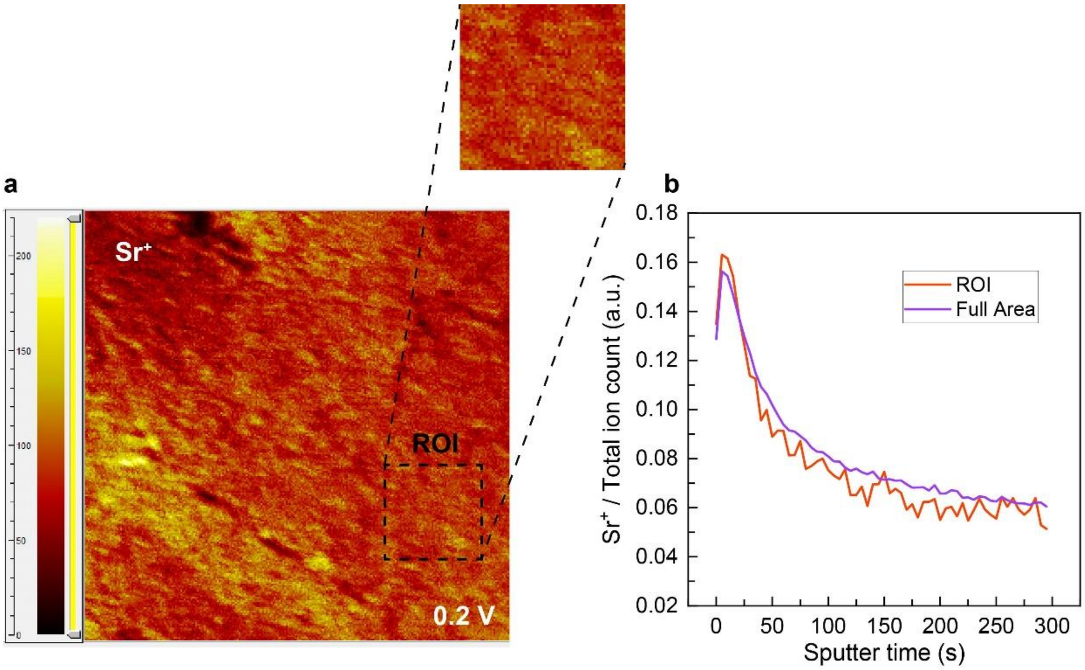

Fig. S9. Lateral uniformity of SIMS 3D profiles. a, top view of Tof-SIMS 3D profiles collected from 0.2 V cathode electrode, the color bar shows count per pixel. **b**, shows depth profiles of Sr^+ using a region of interest (ROI shown by the dashed box) and analysis over the 50×50 μm^2 (Full area). The good agreement between total ion count (TIC) normalized ROI and Full area analysis shows a uniform lateral distribution of ions and a small effect of surface roughness within the analysis area.

a

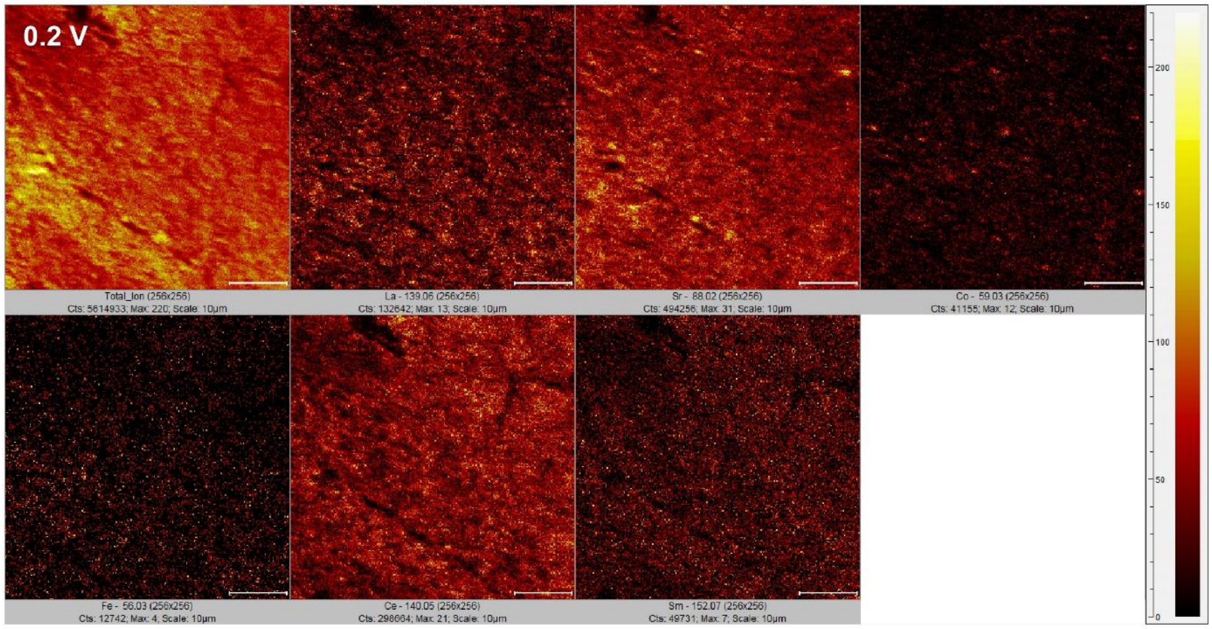

b

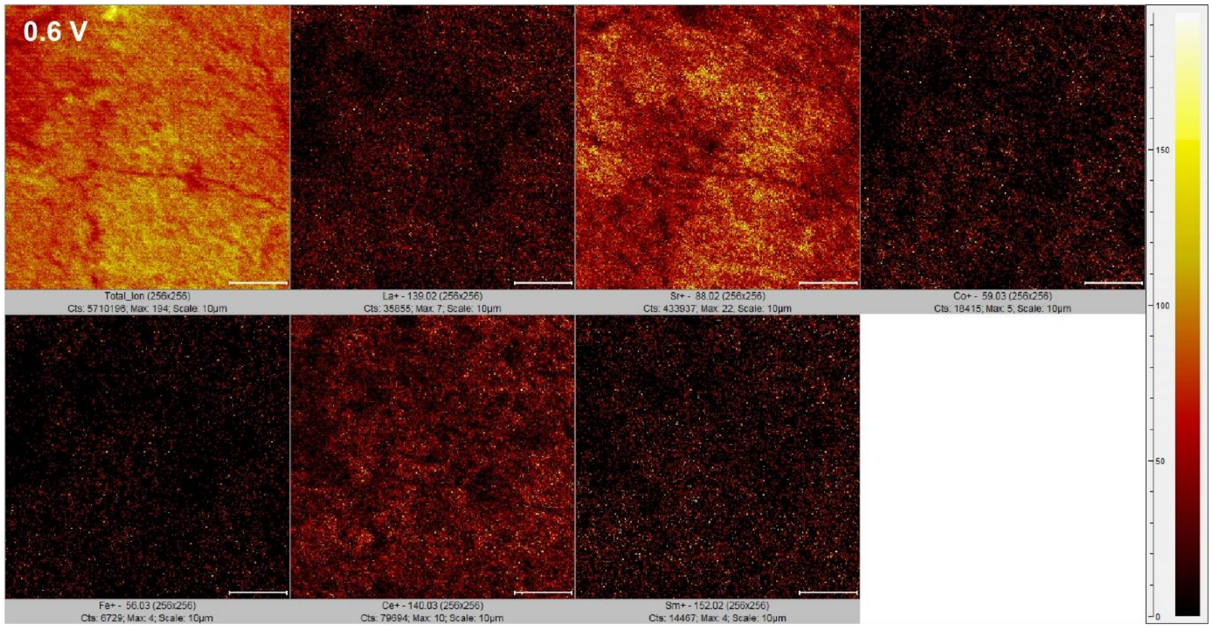

Fig. S10. Integrated 2D SIMS images. Top view of 3D profiles of different elements collected from **a**, 0.2 V and **b**, 0.6 V cathode electrodes. color bars show the count/pixel for each image. The scale bars represent 10 µm. The color bars show count per pixel.

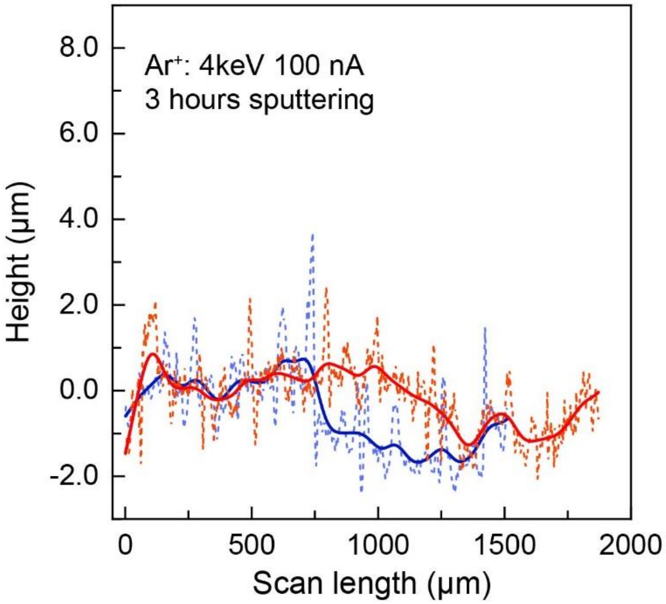

Fig. S11. Mechanical Profilometry. To convert the sputtering rate to sputtering depth a cathode electrode was sputtered using Ar^+ 4 keV, 100 nA ion gun with a 500×500 μm^2 raster size (an identical raster size used to the raster size used for 3D data collection). To reduce the error the sputtering was carried out for 3 hours as opposed to 300 seconds of sputtering which was used for 3D data collection. Due to the high roughness of the cathode surface over the millimeter scan length monitored using Tencor P16+ we used the waviness data (solid line) to calculate the step height. The sputtering rate of 0.15±0.018 nm/s is used for converting sputter time to sputter depth.

SIMS is a matrix-sensitive technique, meaning although we can use the technique to present chemical gradients in the depth profilometry measurements of our samples, ion yields, and subsequent ion counts are functions of other elements and their composition in the matrix, as well as charging effects, especially in areas close to the surface. This makes SIMS measurements presented in this study a semiquantitative analysis as opposed to the quantitative XPS technique used here. That being said, although surface charging and composition effects can impact the semiquantitative B/A ratio presented here, one should expect that such effects have lesser impacts on the B/A ratio below the surface as the bulk regions are more comparable in these samples in terms of composition and charging. To further compare the chemical changes between the samples tested at cathodic overpotentials of -0.35 V and -0.43 V, we took the integral of the curves (highlighted red and green regions in Fig S. 12) with a plateau part of the profiles (region III) as the baseline. As can be seen, the integrated depleted area for the -0.43 V sample is over two times larger than the area extracted for the -0.35 V sample, 9.46 vs. 4.25, respectively. These results

show that -0.43 V potential leads to more depletion of B-site cation below the surface compared to -0.35 V. Due to mass conservation, it is also expected to see more B-site and less A-site cations on the surface of the -0.43 V sample in the absence of external effects such as charging. Hence, we associate the observed larger chemical change with the charging effects in this sample with -0.35 V overpotential. For instance, the presence of residual gold contact after removing the gold contacts from the surface of samples could also contribute to different charging effects between -0.35 V and -0.43 V samples in SIMS measurements. The combination of a larger sub-surface integrated B/A area in SIMS, plus the quantitative XPS analysis confirms more B-site cation exsolution on the surface of -0.43 V compared to -0.35 V.

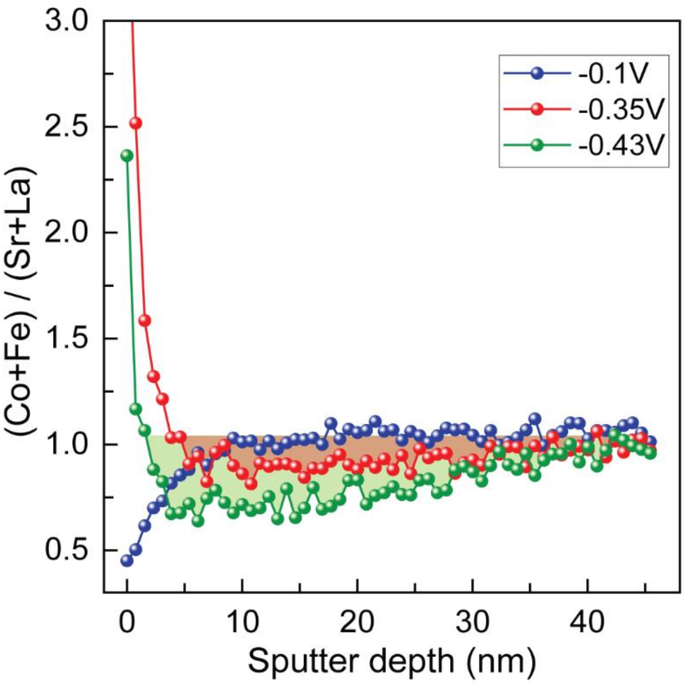

Fig. S12. **1D SIMS profiles of B/A elements**. Semiquantitative depth profile of (Co+Fe)/(La+Sr) for the LSCF-SDC electrodes polarized at -0.1 V, -0.35 V, and -0.43 V. The highlighted regions show the integrated area under the plateau line. The larger absolute integrated region for the -0.43 V sample (-9.46 au.nm) compared to the -0.35 V sample (-4.25 au.nm) shows the larger depletion of the B cite cations in the -0.43 V sample.

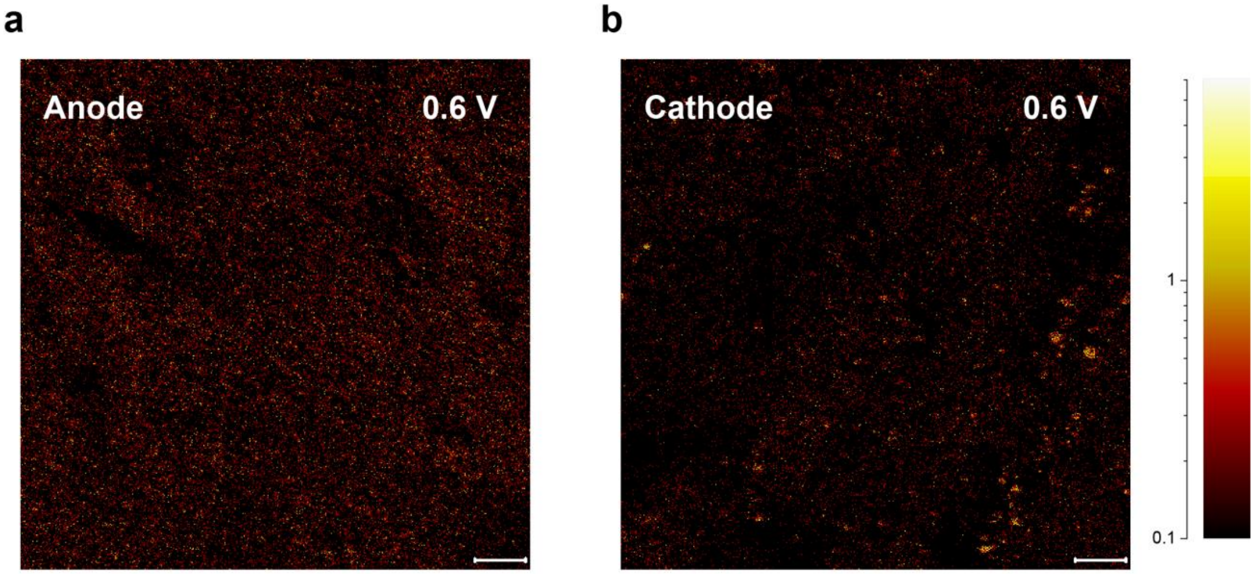

Fig. S13. **2D SIMS images of Co^+ count on anode versus cathod electrodes**. High-resolution 2D SIMS images in unbunched mode. Co^+ (TIC) image of **a**, anode electrode of 0.6 V cell **b,** cathode electrode of 0.6 V cell. The scale bars show 10 µm and the color bar represent count per pixel on each image.

Preferential orientation of RP3 phase

To further study the preferred orientation of RP3 phase formation in different samples, we have also measured the x-ray diffraction pattern for 1 V LSCF-SDC cell applied polarization (resulting in -0.56 overpotential on the cathode). Fig. S14 shows the comparison between cathodically polarized electrodes and the resulting RP3 peaks and cobalt metal peaks. As can be seen the preferential orientation of RP3 phase results in the absence/presence of some peaks in the X-ray diffraction pattern.

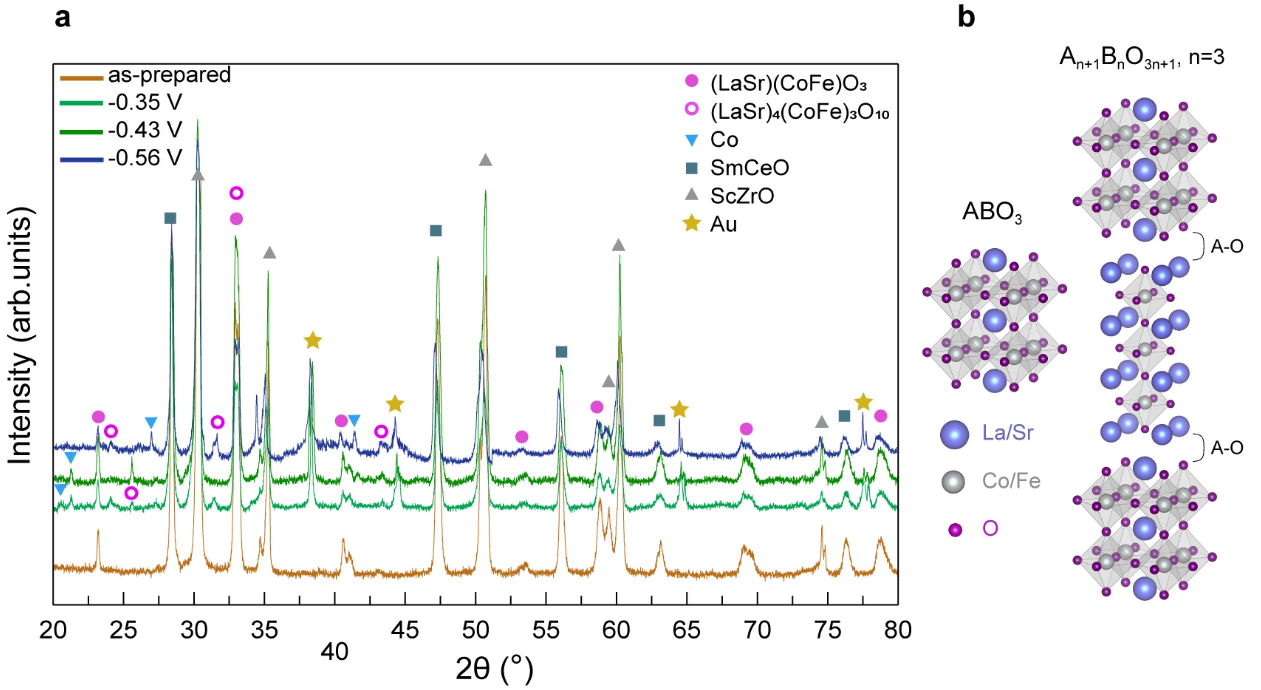

Fig. S14. XRD pattern of LSCF-SDC electrodes upon applied cathodic potentials. a. Ex-situ x-ray diffraction pattern is obtained after quenching the cells from 800° C to room temperature at each applied potential. The diffraction pattern is measured on the cathode electrodes. **b.** Schematic of the crystal structures of the P phase and RP3 phase.

Electrochemical and chemical stability of LSCF

The electrochemical performance of LSCF (without SDC mixed composite) is stable under similar conditions as of which LSCF:SDC shows stability. The polarization resistance and current density of LSCF at 0.2 V and 0.6 V are shown in Fig. S15. As can be seen, LSCF current density is increasing and polarization resistance is decreasing which indicates activation whereas the opposite trend is observed when polarizing the LSCF at 0.2 V, showing degradation. To understand the mechanism behind this activation, we perform surface and structural characterization. Fig. S16 shows the x-ray diffraction pattern of LSCF cell polarized at 0.6 V and as-prepared LSCF. Additional peaks formed in LSCF-0.6V diffraction patterns are assigned to $A_4B_3O_{10}$ (RP3, n=3 in $A_{n+1}B_nO_{3n+1}$) or Ruddlesden-Popper (RP3) perovskite. In addition, several peaks corresponding to cobalt metal appear for the LSCF-0.6V.

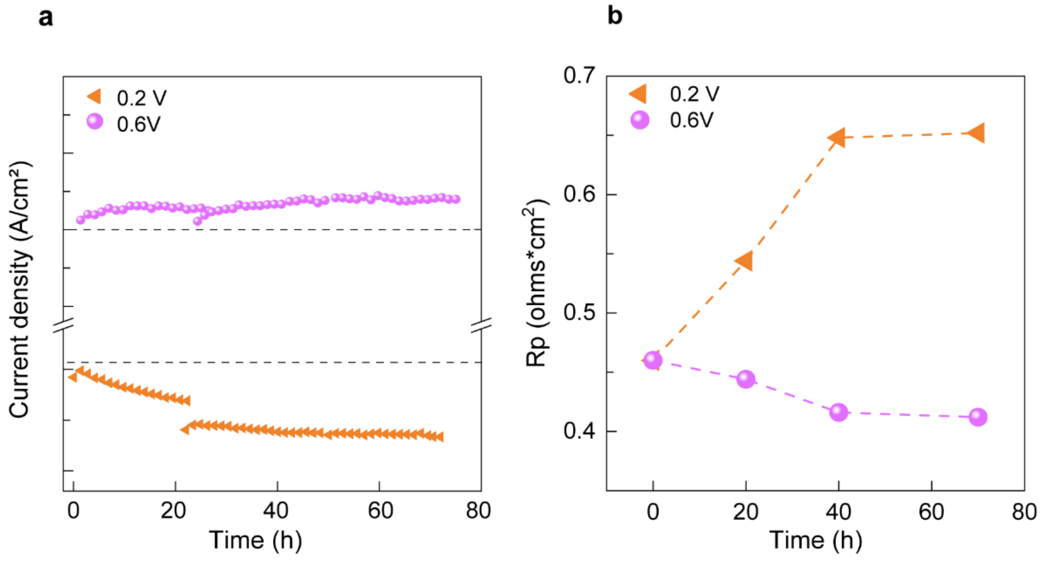

Fig. S15. Stability of the LSCF electrode as a function of electrochemical polarization potential. a, Current density from the symmetric cells measured at corresponding potentials, dashed lines are plotted as a guide for the eye to show how the current density changes with respect to the initial state at that potential. **b**, Polarization resistance obtained from electrochemical impedance spectroscopy at open-circuit voltage as a function of time measured after polarizing the symmetric cell at different potentials.

To understand the relation between RP3 phase formation and surface chemistry, we performed XPS on the surface of LSCF-0.6V. Table. S1 shows the Sr surface and lattice component normalized to total A-site cations and B-site to A-site cation ratios. The Sr surface and lattice

components decrease and increase respectively on the surface of LSCF-0.6 V electrode compared to LSCF. Also Co and Fe precipitate on the surface of LSCF-0.6 V due to the formation of RP3 phase.

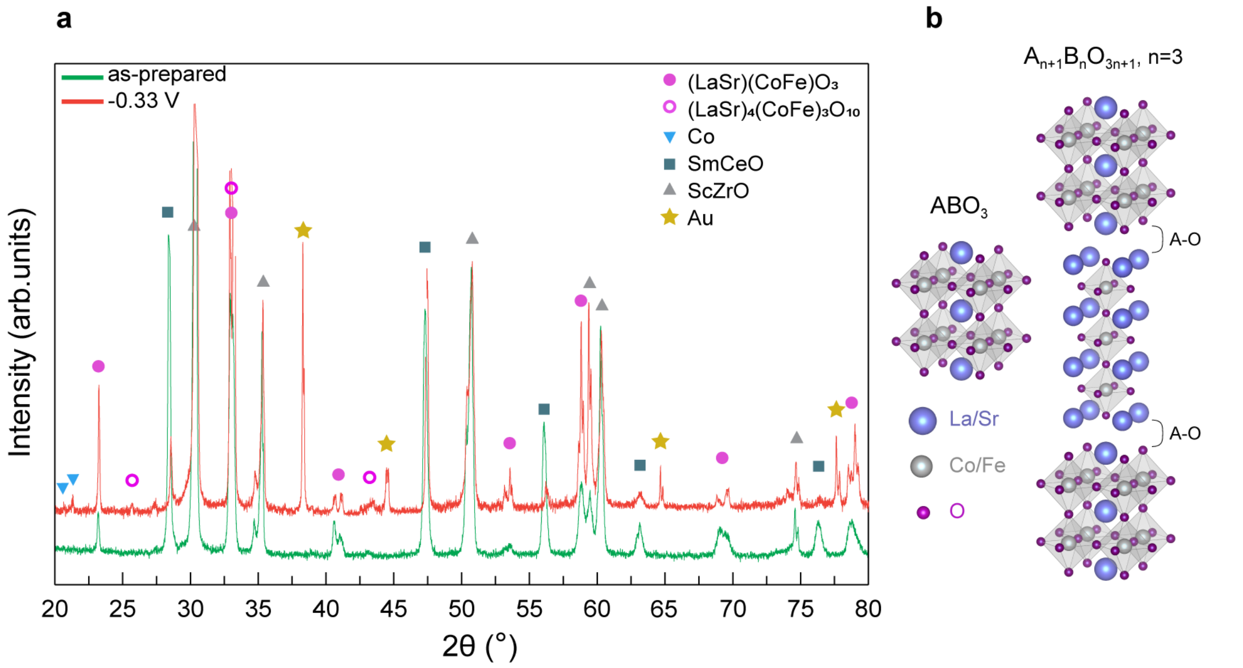

Fig. S16. XRD pattern of LSCF electrodes upon applied cathodic potential. a, Ex-situ x-ray diffraction pattern is obtained after quenching the cells from 800° C to room temperature at each applied potential. The diffraction pattern is measured on the cathode electrode. **b**, Visualization of the crystal structures of the P phase and RP3 phase (VESTA software is used for visualization)[3].

Electrode	(Co+Fe)/(La+Sr)	Sr surface/(Sr+La)	Sr lattice/(Sr+La)
LSCF-0.6V	0.82	0.35	0.11
LSCF	0.61	0.45	0.1

Table. S1. Surface chemistry of polarized and as-prepared LSCF. Surface and lattice concentrations of Sr normalized to the total A-site and the ratio of B-site cations to A-site cations on the surface of LSCF-0.6 V and LSCF.

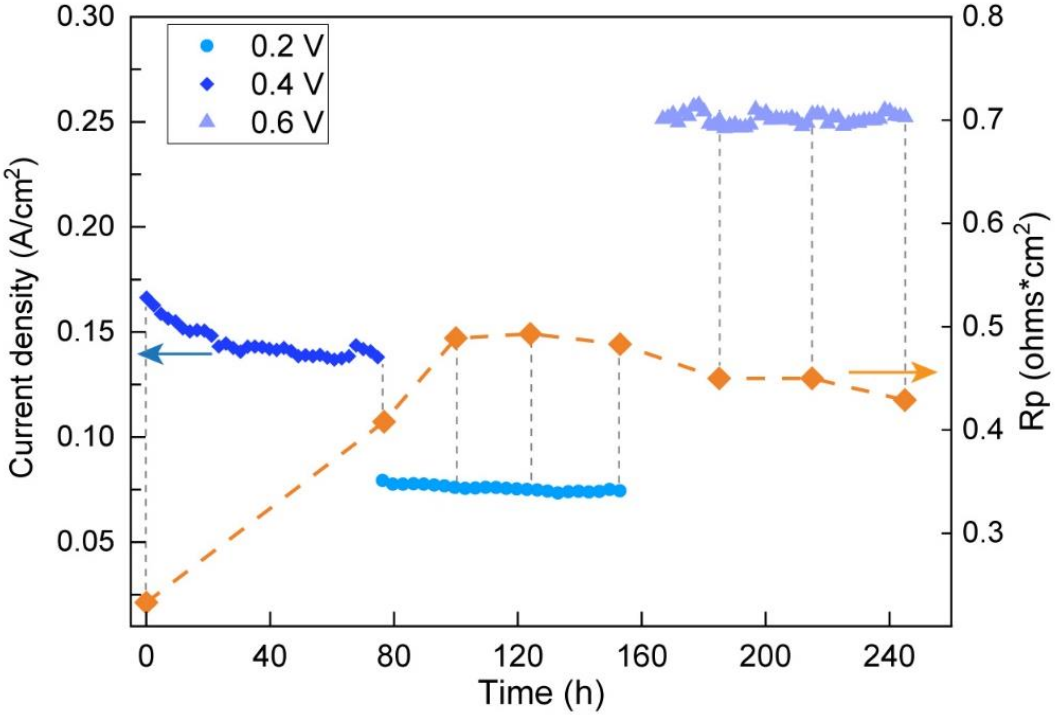

Fig. S17. Electrochemical performance of the fuel cell upon different applied potentials. Current density as a function of time at different applied voltages and fuel electrode (NCS) polarization resistance at OCV after applying each voltage for 80 hours.

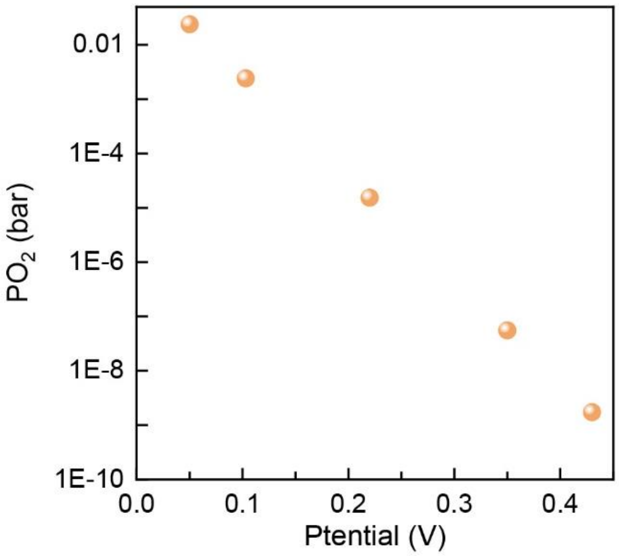

Fig. S18. Oxygen partial pressure as a function of applied cathodic potential. Oxygen partial pressure calculated in logarithmic scale for different cathodically polarized LSCF-SDC cells

Cross-section morphology of LSCF-SDC

Scanning electron microscopy is used to investigate the morphology of the cross-section of as-prepared and cathodically polarized $La_{0.8}Sr_{0.2}Co_{0.8}Fe_{0.2}O_3$-$Sm_{0.2}Ce_{0.8}O_{1.9}$ (SDC) on ScSZ ($Sc_{0.06}Zr_{0.94}O_{1.97}$) electrolyte. Fig. S19. There are no evident morphological changes and delamination in 0.2 V and 0.6 V cells compared to each other and compared to as-prepared cells.

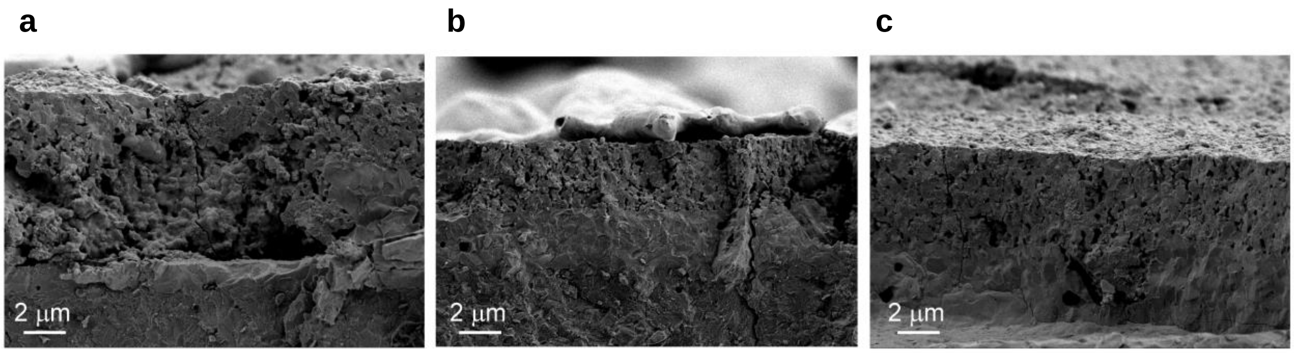

Fig. S19. SEM images of LSCF cross-section. a, as-prepared electrode. **b** polarized at 0.2 V electrode **c**, polarized at 0.6 V electrode

To investigate a possible overlap between Sr *3d* core level spectra and Ce *4d* core level spectra, we measured Ce *4d* from the SDC powder within the binding energy of Sr *3d*. There are no Ce *4d* peaks in this binding energy region.

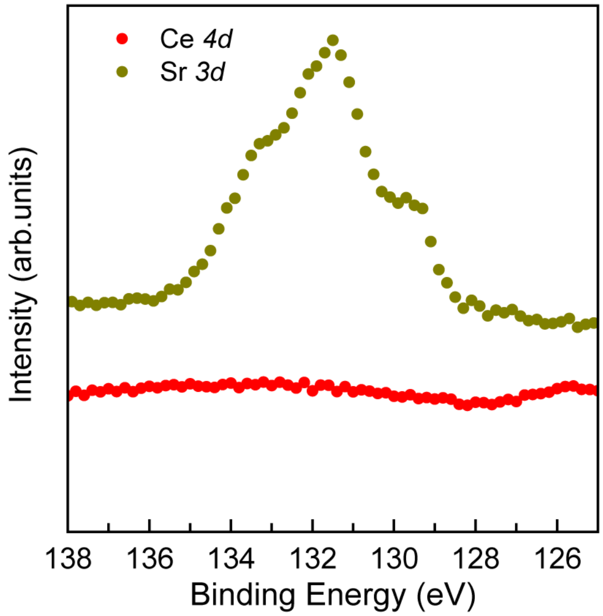

Fig. S20. X-ray photoelectron spectroscopy of Sr *3d* spectra and Ce *4d* spectra.

Supplementary References

1. Fu, C., Sun, K., Zhang, N., Chen, X. & Zhou, D. Electrochemical characteristics of LSCF–SDC composite cathode for intermediate temperature SOFC. *Electrochimica Acta* **52**, 4589-4594 (2007).
2. Ni, N. et al. Degradation of (La0. 6Sr0. 4) 0.95 (Co0. 2Fe0. 8) O3− δ solid oxide fuel cell cathodes at the nanometer scale and below. *ACS applied materials & interfaces* **8**, 17360-17370 (2016).
3. Momma, K. & Izumi, F. VESTA 3 for three-dimensional visualization of crystal, volumetric and morphology data. *Journal of applied crystallography* **44**, 1272-1276 (2011).
