# Supplementary material for: Improvement of oxygen reduction activity and stability on a perovskite oxide surface by electrochemical potential
Source: Nat Commun. 2023 Nov 8;14:7203. doi: 10.1038/s41467-023-42462-5 (PMC10632449; doi:10.1038/s41467-023-42462-5)
Supplement: Supplementary file 2 — Peer Review File [file 41467_2023_42462_MOESM2_ESM.pdf]

nature portfolio

Peer Review File

Improvement of Oxygen Reduction Activity and Stability on a Perovskite Oxide Surface by Electrochemical Potential

Open Access This file is licensed under a Creative Commons Attribution 4.0 International License, which permits use, sharing, adaptation, distribution and reproduction in any medium or format, as long as you give appropriate credit to the original author(s) and the source, provide a link to the Creative Commons license, and indicate if changes were made. In the cases where the authors are anonymous, such as is the case for the reports of anonymous peer reviewers, author attribution should be to 'Anonymous Referee' followed by a clear attribution to the source work. The images or other third party material in this file are included in the article's Creative Commons license, unless indicated otherwise in a credit line to the material. If material is not included in the article's Creative Commons license and your intended use is not permitted by statutory regulation or exceeds the permitted use, you will need to obtain permission directly from the copyright holder. To view a copy of this license, visit http://creativecommons.org/licenses/by/4.0/.

REVIEWER COMMENTS

Reviewer #1 (Remarks to the Author):

The authors analyzed the stability and the improvement of ORR by applying various electrochemical potentials. Results and discussion in this manuscript showed obvious change of stability within 80h testing window. And other characterization techniques were also employed to explain observed improvement with the surface and sub-surface elemental changes. However, results and discussions are not sufficient or strong enough to support their conclusions and the novelty can't be sufficiently justified to be published in Nature Communications. Detailed comments are listed as follow:

1. The authors stated at Page 3, Line 93 that the qualification criteria of a reference electrode setup. However, the description of experimental data at Page 6, Line 132 indicates that the qualification criteria does not actually be met. Please revise related sentences accordingly before the next submission.
2. The description for potentials and overpotentials at different conditions are confusing. The authors are recommended to modify the discussion for better understanding.
3. Page 5, Line 118: In Fig. 1a and 1b, the current density and the Rp showed different tendencies. For instance, when the authors claimed Rp reached a plateau after the first 20h test. However, the current density showed mono tonic increase during the whole testing period.
4. Page 10, Line 200: In Fig. 3, the authors tried to employ SIMS analysis to show the surface chemistry change under electrochemical potentials. In Fig. 3e, the one under -0.35 V shows larger elemental change compared to the one under -0.43 V, which is not consistent to the statement at Page 9, Line 197.
5. Page 12, Line 241: In Fig. 5, peaks labeled for different phases are inconsistent. For example, $(LaSr)_4(CoFe)_3O_{10}$ was labeled in different positions in patterns for ones under -0.35 V and -0.43 V. Please revise or explain these differences.
6. Overall, the stability and the electrochemical performance (Rp changes) have been shown to be improved by applying higher voltages. However, the performance improvements should be demonstrated under practical conditions rather than using the symmetric cell in ambient air.

Reviewer #2 (Remarks to the Author)

This study selected La1-xSrxCo1-xFexO3 (LSCF) -Sm0.2Ce0.8O1.9 (SDC) composite material as the research object, and controlled the surface precipitation phase by changing the reduction electrode potential. It was quantitatively determined that Co and Ruddlesen-Popper-type perovskite structures can be precipitated on the electrode surface when the potential is greater than 0.6V, thereby suppressing the segregation of Sr during the operation and achieving the goal of stabilizing the electrode's electrochemical reduction activity. The determination of surface composition and structure was performed through X-ray photoelectron spectroscopy (XPS), time of flight-secondary ion mass spectrometry (ToF-SIMS), and XRD. The reduction potential loading method selected for this study is a common method for modifying solid oxide fuel cells, and the innovation lies in the discovery that a higher reduction potential can achieve in-situ synthesis of active surface phases. After adding some in-depth analysis and characterization, this study can be considered for publication in Nature Communication. Specific suggestions are as follows:

1. Can this strategy also implement in-situ regulation of similar phases in La1-xSrxCo1-xFexO3 (LSCF) alone? Is it suitable for use in other A-site perovskite cathode materials containing Sr?
2. The RP phase generated in situ on the surface was only identified through XRD in the article, and it is suggested that the authors provide more structural characterization for confirmation, such as high-resolution electron microscopy.
3. It is recommended to prepare a single-cell test to provide data on the electrochemical stability and improvement of the cells after reduction treatment.

We thank the reviewers for their time and thoughtful review of our paper. In response to the constructive comments of all the reviewers and our goal to probe deeper, we have added substantial new data and presented improved activity and stability in full cells. The modified sections in the main text and supporting information can be seen in red colored text.

Specifically, we have now:

- Directly shown that applied electrochemical polarization results in the improvement of fuel cell performance under operational conditions. By using a composite fuel electrode made of NiO and $Sm_{0.2}Ce_{0.8}O_{1.9}$ (abbreviated as NCS), and a composite oxygen electrode made of LSCF-SDC, we have demonstrated that the oxygen reduction reaction activates when increasing the cell voltage from 0.2 V to 0.6 V. In addition to the improved oxygen reduction reaction rate, we observed activation of the fuel electrode and an increase in the maximum power density of the fuel cell after applying a 0.6 V cell voltage.
- Included the validation criteria used in placement of reference electrode. The measurement of polarization resistance with three-electrode configuration and two-electrode configuration are in agreement with each other,
- Resolved the reason behind the apparent differences observed in X-ray diffraction patterns by including additional results. By comparing the X-ray diffraction patterns, we associate this difference with the preferential orientation of RP3 phase formation and the polycrystalline nature of these samples.
- Confirmed that applied electrochemical polarization improves the activity and stability of oxygen reduction reaction in LSCF (without SDC). The applied polarization in LSCF forms RP3 phase and metal cobalt, based on X-ray diffraction patterns. In addition, Sr segregation has been suppressed on the surface of LSCF. The changes in the surface chemistry and structure of LSCF as a result of electrochemical polarization, improve the electrochemical performance in these cells.

Reviewer #1 (Remarks to the Author):

The authors analyzed the stability and the improvement of ORR by applying various electrochemical potentials. Results and discussion in this manuscript showed obvious change of stability within 80h testing window. And other characterization techniques were also employed to explain observed improvement with the surface and sub-surface elemental changes. However, results and discussions are not sufficient or strong enough to support their conclusions and the novelty can't be sufficiently justified to be published in Nature Communications. Detailed comments are listed as follow:

1) The authors stated at Page 3, Line 93 that the qualification criteria of a reference electrode setup. However, the description of experimental data at Page 6, Line 132 indicates that the qualification criteria does not actually be met. Please revise related sentences accordingly before the next submission.

To validate the qualification criteria in the placement of the reference electrode, the sum of polarization resistances of the cathode and anode, in the three-electrode measurement, should be equal to the polarization resistance measured across the cell in the two electrode measurements. In this work, the qualification criteria have been met for all the cells to ensure that the placement of the reference electrode is correct. However, the overpotential at the cathode and anode is smaller than the total applied voltage regardless of the reference electrode position. This is associated with the potential loss across the electrolyte and current collector. In order to clarify the polarization resistance measurement by three electrodes and two electrodes configuration, we have added the following section in the SI on page 2 and revised the main text on pages 4 and 6 to reflect this.

Validation of reference electrode measurement

We compare the polarization resistance measured by two and three electrode configurations to validate the correct placement of the reference electrode. The newly added **Fig. S1** shows the polarization resistance measured by the three electrodes (*Rp1* and *Rp2*) and two electrodes (*Rp* total) configurations. The sum of *Rp1* and *Rp2* (0.37 Ωcm^2 + 0.32 Ωcm^2=0.69 Ωcm^2) equals *Rp* total (0.69 Ωcm^2) which validates the required criteria in placement of the reference electrode.

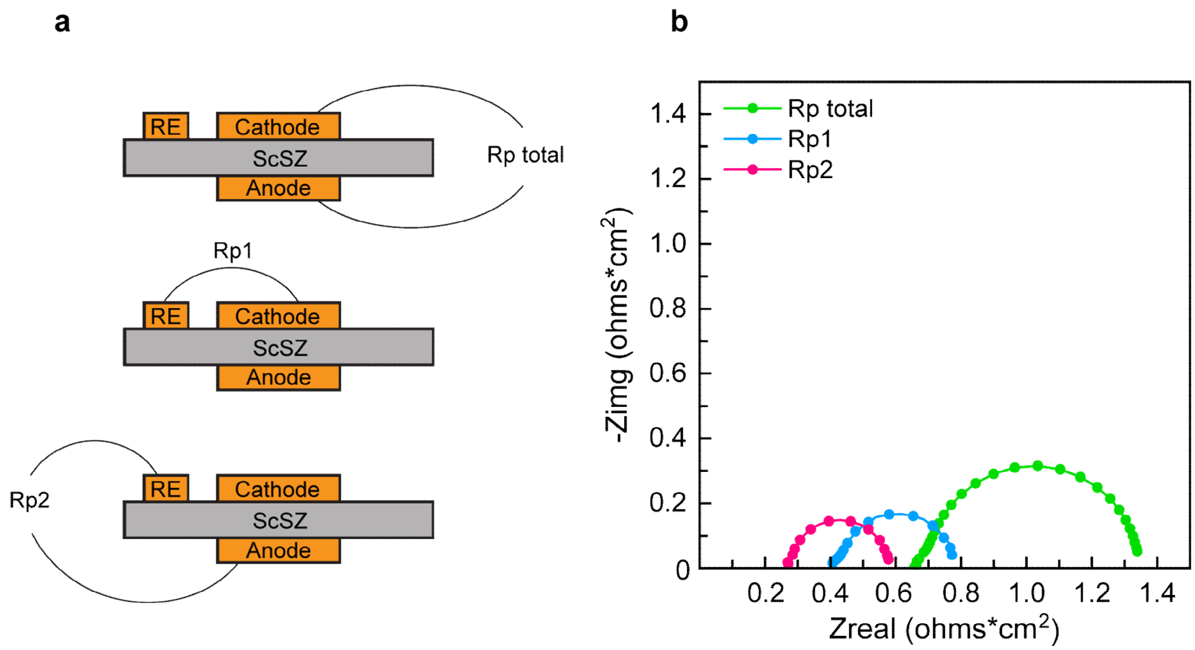

Fig. S1. Polarization resistance measured by two and three electrodes configurations. a, Schematic of the two electrodes (top) and three electrodes (middle and bottom) measurement. **b,** *Rp* total is measured across the cell by using the two electrodes configuration. *Rp1* and *Rp2* are measured by three electrode configurations representing the cathodic and anodic electrodes respectively.

2) The description for potentials and overpotentials at different conditions are confusing. The authors are recommended to modify the discussion for better understanding.

We have two different sets of voltages. The first type is the total applied voltage or total applied polarization across the cell, and the second type is the overpotential on the cathode and anode which is also distinguished by a positive (+) or negative (-) sign. We have revised the text on pages 4, 5, 6, 7, 10, and 12. to reflect this and refer to total the applied potential as "applied cell voltage". In the case of measured potential on cathode and anode by using reference electrode, we refer to this as the "electrode overpotential".

3) Page 5, Line 118: In Fig. 1a and 1b, the current density and the Rp showed different tendencies. For instance, when the authors claimed Rp reached a plateau after the first 20h test. However, the current density showed monotonic increase during the whole testing period.

We agree with the reviewer that the current density and polarization resistance for the 0.6 V and 0.8 V behave differently, and we clarify this in the following section.

Fig. S3 shows the comparison between the *Rp* and current densities for 0.6 V and 0.8 V. At 0.8 V, the cell continues to activate during the 80 hours of operation and an increase in the current density is consistent with a decrease in polarization resistance. The arrows in **Fig. S3 b** indicate the increase in current density with respect to the initial value at time 0 h.

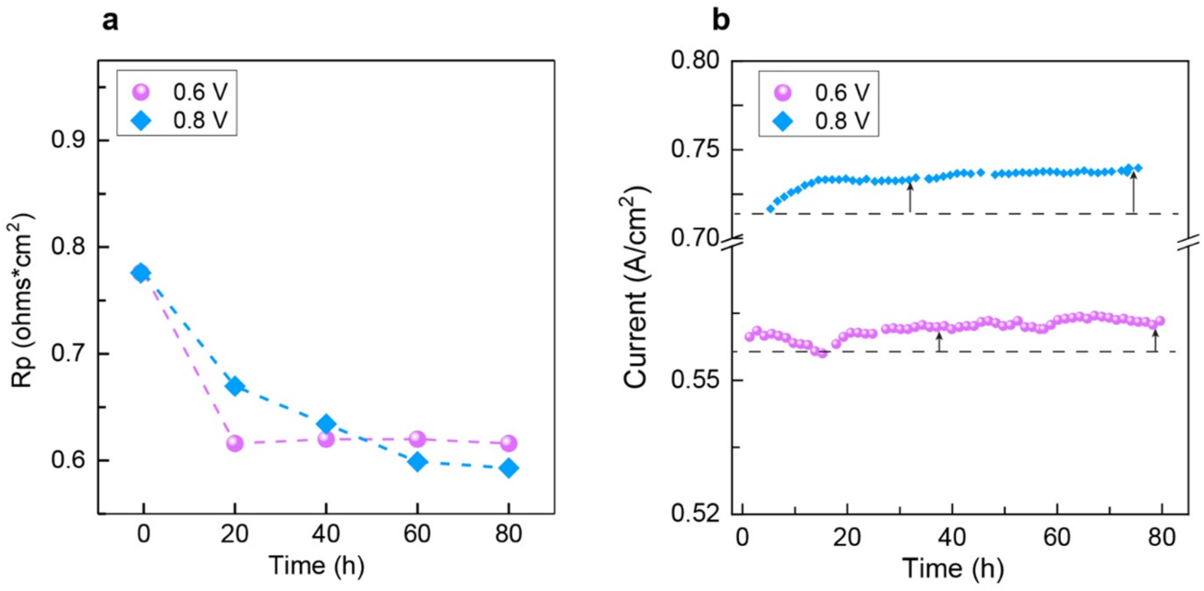

Fig. S3. Comparison of polarization resistance and current density. a, Polarization resistance measured at OCV for 0.6 V and 0.8 v cells. **b,** Current density of 0.6 V and 0.8 V. The arrows indicate the increase in current density with respect to their initial values.

Regarding the 0.6 V cell, the current density between the 60 hours and 75 hours of measurement has a slight increase and plateaus after 75 hours and the current density at 52 hours and 78 hours has similar values. Because there is a smaller increase in current density at 0.6 V compared to 0.8 V (by comparing the arrows in **Fig. S3 b**), the *Rp* of 0.6 V cell shows smaller activation when

compared to the *Rp* of 0.8 V cell. Therefore, the current density and *Rp* behave similarly for each cell but behave differently when comparing the 0.6 V and 0.8 V cells.

We have revised the text on page 4 and SI pages 4 and 5 to reflect this.

4) Page 10, Line 200: In Fig. 3, the authors tried to employ SIMS analysis to show the surface chemistry change under electrochemical potentials. In Fig. 3e, the one under -0.35 V shows larger elemental change compared to the one under -0.43 V, which is not consistent to the statement at Page 9, Line 197.

To answer the reviewer's comment we should note that SIMS is a matrix-sensitive technique, meaning although we can use the technique to present chemical gradients in the depth profilometry measurements of our samples, ion yields and subsequent ion counts are functions of other elements and their composition in the matrix, as well as charging effects especially in areas close to the surface. This makes SIMS measurements presented in this study a semiquantitative analysis as opposed to the quantitative XPS technique used here. That being said, although surface charging and composition effects can impact the semiquantitative B/A ratio presented here, one should expect that such effects have lesser impacts on the B/A ratio below the surface as the bulk regions are more comparable in these samples in terms of composition and charging. To further compare the chemical changes between the samples tested at cathodic overpotentials of -0.35 V and -0.43 V, we took the integral of the curves (highlighted red and green regions in **Fig S. 12**) with a plateau part of the profiles (region III) as the baseline. As can be seen, the integrated depleted area for the -0.43 V sample is over two times larger than the area extracted for the -0.35 V sample, 9.46 vs. 4.25, respectively. These results show that -0.43 V potential leads to more depletion of B-site cation below the surface compared to -0.35 V. Due to mass conservation, it is also expected to see more B-site and less A-site cations on the surface of the -0.43 V sample in the absence of external effects such as charging. Hence we associate the observed larger chemical change with the charging effects in this sample with -0.35 V overpotential. For instance, the presence of residual gold contact after removing the gold contacts from the surface of samples could also contribute to different charging effects between -0.35 V and -0.43 V samples in SIMS measurements. The combination of a larger sub-surface integrated B/A area in SIMS, plus the quantitative XPS analysis confirms more B-site cation exsolution on the surface of -0.43 V compared to -0.35 V. We have modified the text on page 9 of the main text and SI pages 13 and 14 to discuss these points.

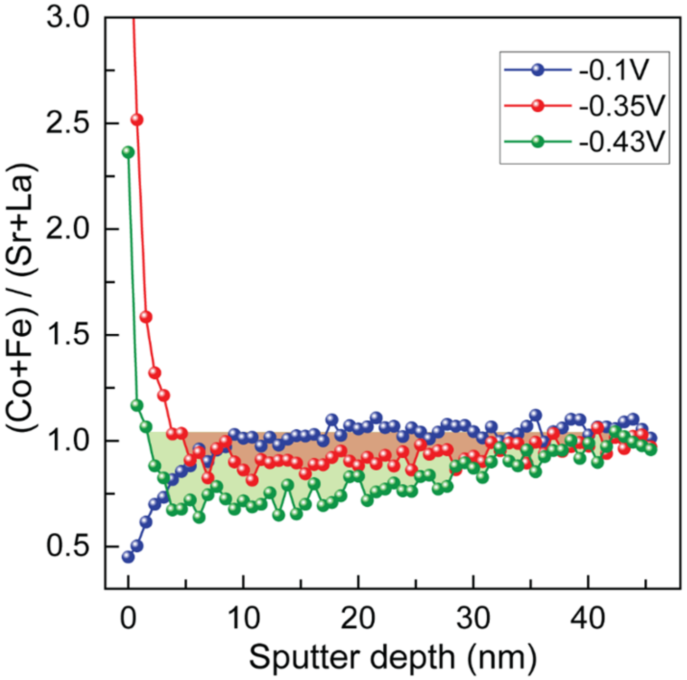

Fig. S12. Semiquantitative depth profile of (Co+Fe)/(La+Sr) for the LSCF-SDC electrodes polarized at -0.1 V, -0.35 V, and -0.43 V. The highlighted regions show the integrated area under the plateau line. The larger absolute integrated region for the -0.43 V sample (-9.46 au.nm) compared to the -0.35 V sample (-4.25 au.nm) shows the larger depletion of the B cite cations in the -0.43 V sample.

5) Page 12, Line 241: In Fig. 5, peaks labeled for different phases are inconsistent. For example, (LaSr)4(CoFe)3O10 was labeled in different positions in patterns for ones under -0.35 V and -0.43 V. Please revise or explain these differences.

To further study the preferred orientation of RP3 phase formation in different samples, we have also measured the X-ray diffraction pattern for 1.0 V LSCF-SDC (resulting in -0.56 overpotential on the cathode). **Fig. S14** shows the comparison between cathodically polarized electrodes and the resulting RP3 peaks and cobalt metal peaks. We observe that peaks related to RP3 and metallic cobalt are not consistent when comparing the X-ray diffraction patterns of different cathodically polarized electrodes.

We have added this section in the supporting information to compare the X-ray diffraction patterns of the electrodes with -0.35 V, -0.43 V, and -0.56 V overpotentials. For all these electrodes

we observe the $(LaSr)_4(CoFe)_3O_{10}$ peaks and metallic cobalt peaks however since these cells are porous polycrystalline, we associate the inconsistency of certain peaks in X-ray diffraction patterns to the preferential orientation of the $(LaSr)_4(CoFe)_3O_{10}$ phase and cobalt in each electrode. We have also revised the text on page 12 and SI on page 16 to explain this difference.

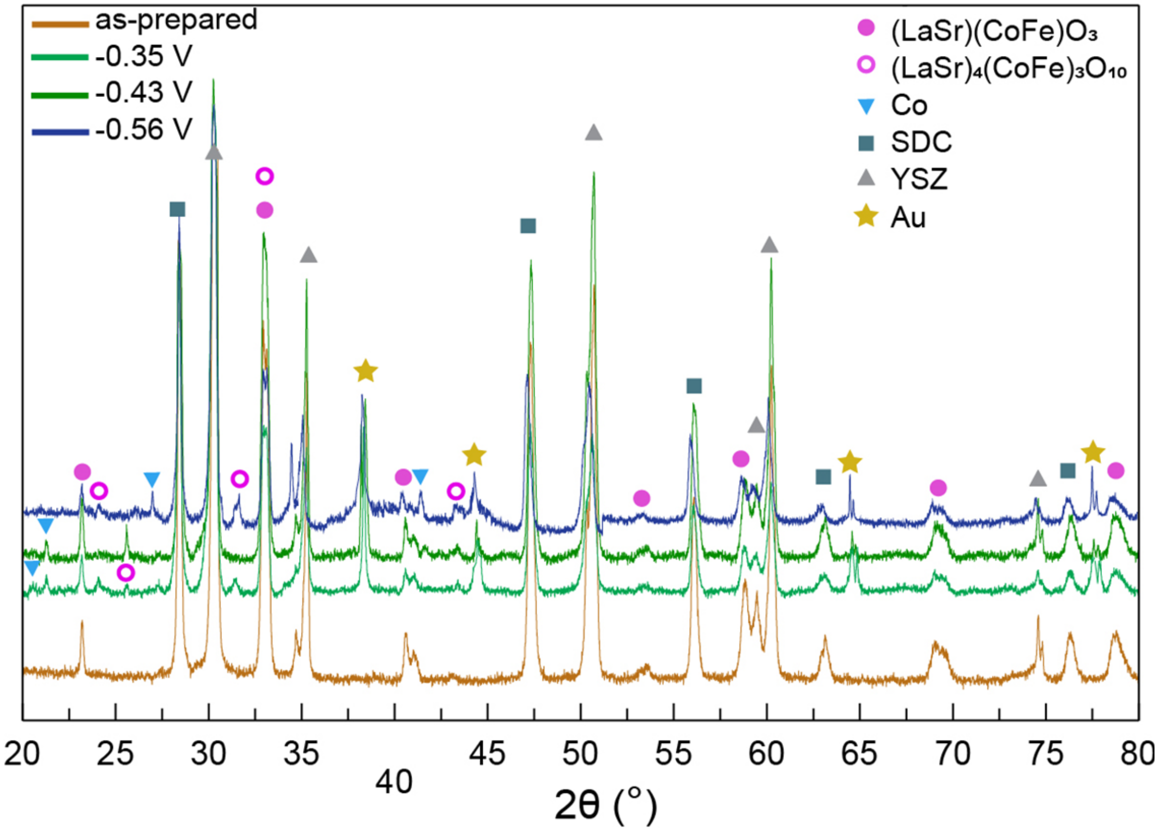

Fig. S14. XRD pattern of LSCF-SDC electrodes upon applied cathodic potentials. Ex-situ X-ray diffraction pattern is obtained after quenching the cells from 800° C to room temperature at each applied potential. The diffraction pattern is measured on the cathode electrodes.

6) Overall, the stability and the electrochemical performance (Rp changes) have been shown to be improved by applying higher voltages. However, the performance improvements should be demonstrated under practical conditions rather than using the symmetric cell in ambient air.

We have added new experiments to investigate the role of applied polarization in the electrochemical activity and stability of full fuel cells. For this experiment, we fabricate fuel cells with NCS composite (NiO | $Sm_{0.2}Ce_{0.8}O_{1.9}$, 70 | 30 ratios by weight) as fuel electrodes and LSCF-SDC composite as oxygen electrodes on ScSZ electrolyte. The electrochemical tests were carried out at 800 °C with 3% H_2 and 3% N_2 as fuel and ambient air as the oxidant under cell voltages of 0.2 V, 0.4 V, and 0.6 V. As can be seen in **Fig. 6**, after applying 0.2 V and 0.4 V, the polarization resistance of the oxygen electrode, measured by the reference electrode, increases, and the current density decreases indicating degradation. On the other hand, by increasing the cell voltage to 0.6 V, the polarization resistance of the oxygen electrode decreases and current density shows stability until the end of the measurement period. Thus, increasing the voltage improves the electrochemical performance of the fuel cell.

The current density-potential-power (j-V-P) curves are measured at the beginning of the experiment (t=0 h) and after applying each cell voltage for 80 hours. When comparing the power densities, we observe that by applying cell voltages of 0.2 V and 0.4 V, the maximum power density (P_{max}, indicated by a red symbol on each curve) decreases compared to the initial P_{max}, however, the P_{max} increases after applying 0.6 V cell voltage, showing activation. It is worth mentioning that the P_{max} after 0.6 V is smaller than P_{max} after 0.4 V and initial P_{max}. The reason behind this drop in P_{max} is the degradation of the fuel electrode polarization resistance. **Fig. S17** shows the polarization resistance of the NCS fuel electrode measured at OCV. The *Rp* of the NCS electrode increases (degradation) by applying 0.2 V and 0.4 V and decreases (activation) by applying 0.6 V. Even though we observe activation of NCS *Rp* by increasing the cell voltage from 0.2 V to 0.6 V, the *Rp* at t=240 h is still larger than t=0 h and t=80 h and some degradation of NCS fuel electrode remains. Based on the observed electrochemical performance of the full fuel cell, Increasing the cell voltage to 0.6 V improves the *Rp* of both fuel and oxygen electrodes, stabilizes current, and results in relative improvement of power density. This experiment confirms that the electrochemical performance of LSCF-SDC oxygen electrode activates and improves at moderate cathodic overpotentials also in a full solid oxide fuel cell under realistic conditions, and not only in the

symmetric cells that we originally presented in our first submission of the manuscript. We have added this new experiment on pages 1, 3, 14, 15, 17, and 18 of the main text and page 19 of SI.

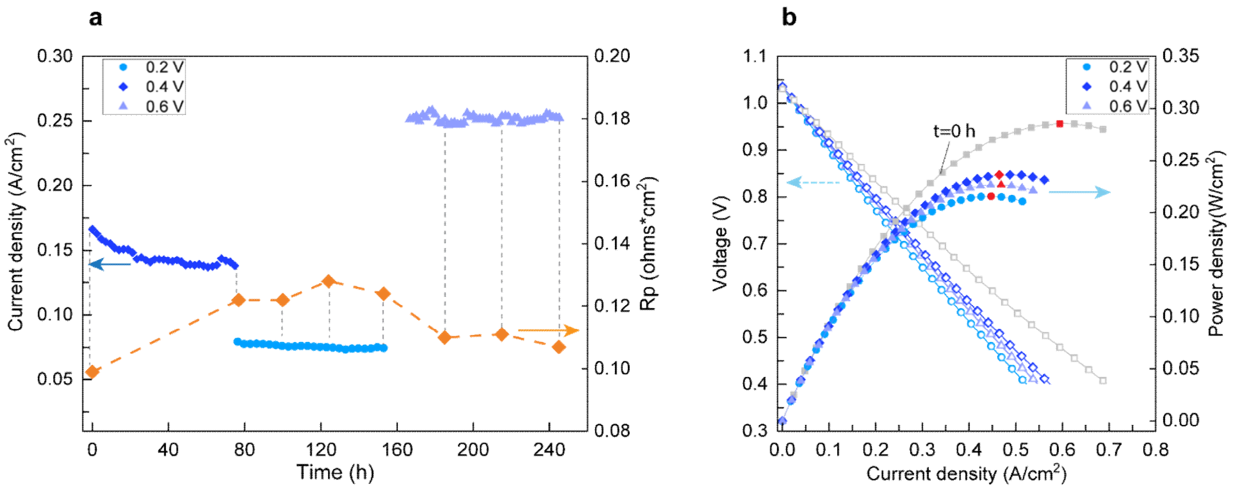

Fig. 6. Electrochemical performance of fuel cell upon different applied potentials. a, current density as a function of time at different applied cell voltages and oxygen electrode polarization resistance at OCV at t=0 h and after applying each voltage for 80 hours. **b,** j-V-P curve of the fuel cell after applying each voltage and at the beginning of the experiment at t=0 h. The maximum power density is indicated by a red symbol on each curve.

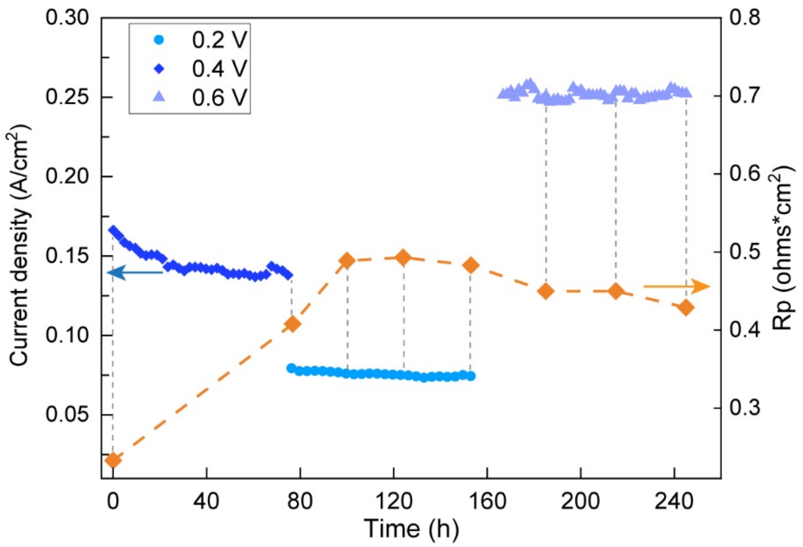

Fig. S17. Electrochemical performance of the fuel cell upon different applied potentials. Current density as a function of time at different applied cell voltages and fuel electrode (NCS) polarization resistance at OCV after applying each voltage for 80 hours.

Reviewer #2 (Remarks to the Author):

This study selected La1-xSrxCo1-xFexO3 (LSCF) -Sm0.2Ce0.8O1.9 (SDC) composite material as the research object, and controlled the surface precipitation phase by changing the reduction electrode potential. It was quantitatively determined that Co and Ruddlesen-Popper-type perovskite structures can be precipitated on the electrode surface when the potential is greater than 0.6V, thereby suppressing the segregation of Sr during the operation and achieving the goal of stabilizing the electrode's electrochemical reduction activity. The determination of surface composition and structure was performed through X-ray photoelectron spectroscopy (XPS), time of flight-secondary ion mass spectrometry (ToF-SIMS), and XRD. The reduction potential loading method selected for this study is a common method for modifying solid oxide fuel cells, and the innovation lies in the discovery that a higher reduction potential can achieve in-situ synthesis of active surface phases. After adding some in-depth analysis and characterization, this study can be considered for publication in Nature Communication. Specific suggestions are as follows:

1) Can this strategy also implement in-situ regulation of similar phases in La1-xSrxCo1-xFexO3 (LSCF) alone? Is it suitable for use in other A-site perovskite cathode materials containing Sr?

As suggested by the reviewer, we further study LSCF (without SDC) by polarizing the cell at 0.2 V and 0.6 V. The electrochemical performance of LSCF (without SDC mixed composite) is stable under similar conditions as which LSCF-SDC shows stability. The polarization resistance and current density of LSCF at 0.2 V and 0.6 V are shown in **Fig. S15**. As can be seen at 0.6 V, LSCF current density is increasing and polarization resistance is decreasing which indicates activation, whereas the opposite trend is observed when polarizing the LSCF at 0.2 V, showing degradation. By applying a cell voltage of 0.6 V and 0.2 V, an overpotential of -0.33 V and -0.1 V have been applied on the cathodic electrodes respectively. To understand the mechanism behind this activation, we perform surface and structural characterization on the cathodic electrode of LSCF. The new **Fig. S16** shows the X-ray diffraction pattern of -0.33 V LSCF electrode and as-prepared LSCF. Additional peaks formed in the -0.33 V LSCF diffraction pattern are assigned to $A_4B_3O_{10}$ (RP3, n=3 in $A_{n+1}B_nO_{3n+1}$) or Ruddlesden-Popper (RP3) perovskite. In addition, several peaks corresponding to cobalt metal appear for the -0.33 V LSCF.

These are consistent with our findings of the cathodically polarized and electrochemically improved LSCF-SDC composite electrode. In addition, XPS analysis shows that the Sr surface and lattice components decrease and increase, respectively, and Co and Fe enrich on the surface of the -0.33 V LSCF electrode compared to the as-prepared LSCF (**Table S1**). Thus the surface chemistry evolution of the cathodically polarized LSCF is similar to that in the LSCF-SDC composite electrode.

We modified the main text on page 15 and SI on pages 18 and 19 with this new experiment and data. These findings on LSCF alone are consistent with the behavior of LSCF-SDC. This means that SDC does not play a significant role in the activation of LSCF under cathodic polarization.

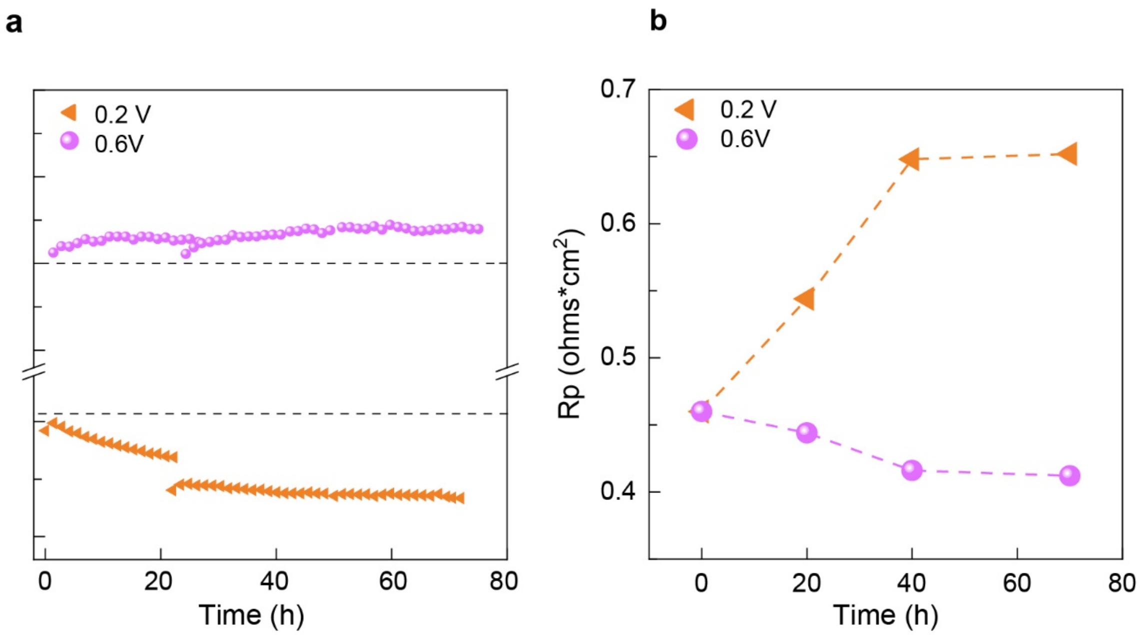

Fig. S15. Stability of the LSCF electrode as a function of electrochemical polarization potential. a, Current density from the symmetric cells measured at corresponding potentials, dashed lines are plotted as a guide for the eye to show how the current density changes with respect to the initial state at that potential. **b**, Polarization resistance obtained from electrochemical impedance spectroscopy at open-circuit voltage as a function of time measured after polarizing the symmetric cell at different potentials.

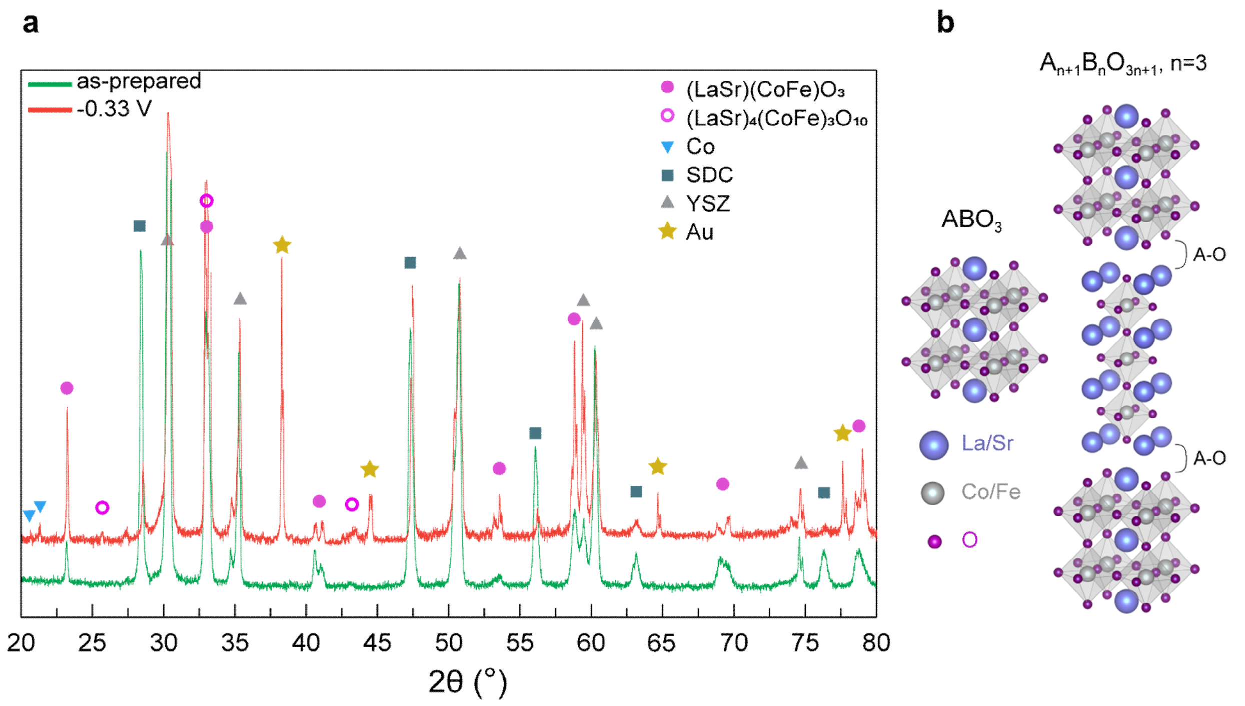

Fig. S16. XRD pattern of LSCF electrodes upon applied cathodic potentials. A, Ex-situ X-ray diffraction pattern is obtained after quenching the cells from 800 °C to room temperature at each applied potential. The diffraction pattern is measured on the cathode electrode. **b**, Visualization of the crystal structures of the P phase and RP3 phase (VESTA software is used for visualization)[1].

Electrode	(Co+Fe)/(La+Sr)	Sr surface/(Sr+La)	Sr lattice/(Sr+La)
-0.33 V LSCF	0.82	0.35	0.11
As prepared LSCF	0.61	0.45	0.1

Table. S1. Surface chemistry of polarized and as-prepared LSCF. Surface and lattice concentrations of Sr normalized to the total A-site and the ratio of B-site cations to A-site cations on the surface of LSCF-0.6 V and LSCF.

To further ascertain the generalizability of our method, we also investigate the role of applied electrochemical polarization in the electrochemical performance of $Sr_{0.7}Ti_{0.3}Fe_{0.7}Co_{0.3}O_3$ (STFC) used as an oxygen electrode in the oxidative coupling of methane. **Fig. R1** shows the X-ray diffraction pattern of STFC polarized at 1.0 V and as prepared STFC. Several additional peaks appear for 1.0 V STFC related to $(ST)_2(FC)_1O_4$ or $A_2B_1O_4$ RP1 phase. As these results are part of an ongoing study on the investigation of the secondary phase formation of STFC in the oxidative coupling of methane, which we are planning to present in a separate study, we did not include

STFC secondary phase formation results in the current paper or supporting information. But we are sharing them here to confirm the general applicability of our findings beyond LSCF-SDC electrodes.

It has also been shown In our previous works that In a thin film of $La_{0.6}Sr_{0.4}FeO_3$, the exsolution of Fe and formation of the RP1 phase result in the improvement of the electronic conductivity of LSF.[2] By using thin film of LSF as a model system in a reducing condition of 0.5 Torr H_2, at 400 °C the exsolution of Fe and formation of RP1 phase take place. So, we believe that the formation of RP phase (n=1 or 3) and exsolution of B-site cations under reducing conditions (by using H_2 flow or by cathodic polarization of the electrode) are applicable in a variety of perovskite oxide systems such as LSCF-SDC, LSCF, STFC, and LSF.

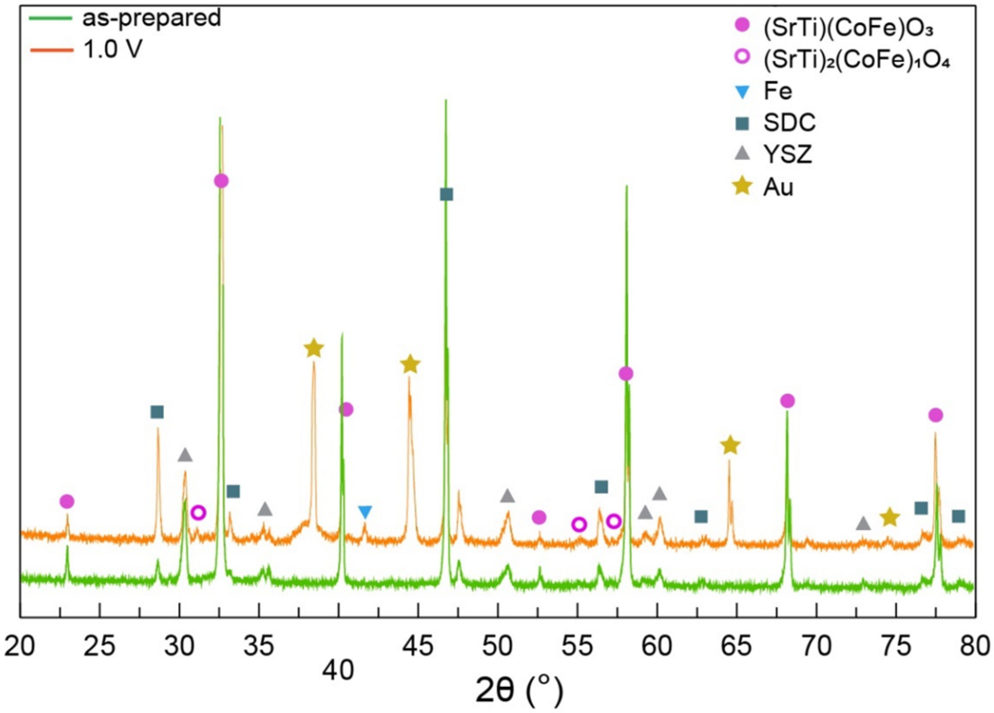

Fig. R1. XRD pattern of STFC electrode upon applied cathodic potentials. Ex-situ x-ray diffraction pattern is obtained after quenching the cells from 800° C to room temperature at 1 V applied cell voltage. The diffraction pattern is measured on the cathode electrodes.

2) The RP phase generated in situ on the surface was only identified through XRD in the article, and it is suggested that the authors provide more structural characterization for confirmation, such as high-resolution electron microscopy.

To acquire high-resolution TEM images, we prepared FIB lamella samples. Unfortunately, despite our several attempts, due to the polycrystalline, high porosity, and rough nature of these samples, we were not able to perform zone-axis alignment for these samples under the electron beam. While the nature of these samples makes the beam alignment challenging, it is reassuring that we previously observed the RP phase formation in high-resolution TEM of thin film LSF samples[2].

3) It is recommended to prepare a single-cell test to provide data on the electrochemical stability and improvement of the cells after reduction treatment.

This important point was also raised by reviewer #1. Please see our answer to the first reviewer's comment#6, presented and discussed on pages 1, 3, 14, 15, 17, and 18 of the main text, on page 19 of SI, and on pages 8 and 9 of this response letter.

References

1. Momma, K.; Izumi, F. *Journal of applied crystallography* **2011,** 44, (6), 1272-1276.
2. Wang, J.; Syed, K.; Ning, S.; Waluyo, I.; Hunt, A.; Crumlin, E. J.; Opitz, A. K.; Ross, C. A.; Bowman, W. J.; Yildiz, B. *Advanced Functional Materials* **2021,** 32, (9).

REVIEWERS' COMMENTS

Reviewer #2 (Remarks to the Author):

The author has replied the corresponding issues properly. Onemore suggestion: it will be expected that the author give more discussion on the in-situ performance since that external potential will constantly influence the microscopic surface struction. Such as, under SOFC and SOEC, the performance should be remarkably different?

Reviewer #3 (Remarks to the Author):

This paper presents some interesting findings on improving the stability and activity of LSCF-SDC oxygen electrodes for solid oxide fuel cells and electrolyzers by applying a cathodic polarization. The results demonstrate that applying a sufficiently high cathodic overpotential (>0.4V) leads to enhanced ORR activity and stability over extended periods of operation. This is attributed to suppression of detrimental Sr surface segregation, formation of exsolved Co nanoparticles, and formation of a Ruddlesden-Popper phase in the near-surface region. Meanwhile, The combination of surface characterization (XPS) and depth profiling (SIMS) provides convincing evidence linking the electrochemical improvements to the changes in surface chemistry and structure. The XPS shows reduced Sr and enhanced Co/Fe at the surface, while the SIMS reveals cation depletion in the near-surface indicative of RP phase formation.

The observations are logically explained through the effective lowering of oxygen partial pressure at the cathode surface under polarization, which enables perovskite decomposition and Co exsolution even in ambient air.

Based on reviewing the attached reply letter, I believe the authors have thoroughly and thoughtfully addressed the reviewers' critiques and suggestions.

Here is my assessment:

The addition of fuel cell data testing the approach under real operating conditions is a major strength. It convincingly shows the benefits of the cathodic polarization on oxygen electrode performance in a full cell. The results align well with the symmetric cell data.

The validation of the reference electrode setup helps justify that measurement approach.

Showing the 2-electrode and 3-electrode results match is an important control.
Explaining the preferential RP phase orientation provides a reasonable rationale for the inconsistencies in the XRD patterns between samples.
Testing on LSCF without SDC helps isolate the effect and show it does not rely on the SDC phase. The surface chemistry analyses confirm similar impacts as the composite.
The SEM imaging attempts, though unsuccessful, show effort to acquire higher resolution structural data as suggested.
The discussion of matrix effects in SIMS helps account for the apparent disagreement in elemental ratios between the -0.35V and -0.43V samples. The integrated SIMS data support the XPS trends.
Showing the applicability to other perovskites (STFC) indicates this is likely a general phenomenon, not just specific to LSCF-SDC.
Overall, I believe the authors have been very responsive to the critiques, and the revisions significantly strengthen the work. The fuel cell data in particular provide an important validation of the approach under operating conditions. The reviewers' concerns appear to be adequately addressed, and I think this response letter makes a compelling case that their manuscript merits publication. Provided the revisions are incorporated in the paper itself, the response seems comprehensive and satisfactory in my assessment.

We thank the reviewers for their positive and constructive review of our paper. In response to the reviewers' comments, we have modified the main text which can be seen in red colored text. Below are the reviewers' comments and our point-by-point response.

Reviewer #2 (Remarks to the Author):

The author has replied the corresponding issues properly. Onemore suggestion: it will be expected that the author give more discussion on the in-situ performance since that external potential will constantly influence the microscopic surface struction. Such as, under SOFC and SOEC, the performance should be remarkably different?

We thank the reviewer for the suggestion. The full cell tests were carried out in SOFC mode where the oxygen electrode was cathodically polarized and is the focus of this study. The reason is that the cathodic electrode shows a larger extent of chemical and electrochemical improvement compared to the anodic electrode. The dominant changes in the cathodic electrode can be seen from how much the Rp changes as shown in Fig. 1c and Fig. 1d, and the near-surface chemistry evolution in Fig. 2 and Fig. S6. In addition, the formation of the RP3 phase and exsolution of Co only happen on the cathodic electrode.

We have modified the main text on page 11 to reflect this point.

Reviewer #3 (Remarks to the Author):

This paper presents some interesting findings on improving the stability and activity of LSCF-SDC oxygen electrodes for solid oxide fuel cells and electrolyzers by applying a cathodic polarization. The results demonstrate that applying a sufficiently high cathodic overpotential (>0.4V) leads to enhanced ORR activity and stability over extended periods of operation. This is attributed to suppression of detrimental Sr surface segregation, formation of exsolved Co nanoparticles, and formation of a Ruddlesden-Popper phase in the near-surface region. Meanwhile, The combination of surface characterization (XPS) and depth profiling (SIMS) provides convincing evidence linking the electrochemical improvements to the changes in surface chemistry and structure. The XPS shows reduced Sr and enhanced Co/Fe at the surface, while the SIMS reveals cation depletion in the near-surface indicative of RP phase formation.

The observations are logically explained through the effective lowering of oxygen partial pressure at the cathode surface under polarization, which enables perovskite decomposition and Co exsolution even in ambient air.

Based on reviewing the attached reply letter, I believe the authors have thoroughly and thoughtfully addressed the reviewers' critiques and suggestions.

Here is my assessment:

The addition of fuel cell data testing the approach under real operating conditions is a major strength. It convincingly shows the benefits of the cathodic polarization on oxygen electrode performance in a full cell. The results align well with the symmetric cell data.

The validation of the reference electrode setup helps justify that measurement approach. Showing the 2-electrode and 3-electrode results match is an important control.

Explaining the preferential RP phase orientation provides a reasonable rationale for the inconsistencies in the XRD patterns between samples.

Testing on LSCF without SDC helps isolate the effect and show it does not rely on the SDC phase. The surface chemistry analyses confirm similar impacts as the composite.

The SEM imaging attempts, though unsuccessful, show effort to acquire higher resolution structural data as suggested.

The discussion of matrix effects in SIMS helps account for the apparent disagreement in elemental ratios between the -0.35V and -0.43V samples. The integrated SIMS data support the XPS trends.

Showing the applicability to other perovskites (STFC) indicates this is likely a general phenomenon, not just specific to LSCF-SDC.

Overall, I believe the authors have been very responsive to the critiques, and the revisions significantly strengthen the work. The fuel cell data in particular provide an important validation of the approach under operating conditions. The reviewers' concerns appear to be adequately addressed, and I think this response letter makes a compelling case that their manuscript merits publication. Provided the revisions are incorporated in the paper itself, the response seems comprehensive and satisfactory in my assessment.

We appreciate reviewer #3's highly positive comments in response to our resubmission and their valuable assessment.
